# Patterns of Sequence Divergence and Evolution of the *S_1_* Orthologous Regions between Asian and African Cultivated Rice Species

**DOI:** 10.1371/journal.pone.0017726

**Published:** 2011-03-10

**Authors:** Romain Guyot, Andrea Garavito, Frédérick Gavory, Sylvie Samain, Joe Tohme, Alain Ghesquière, Mathias Lorieux

**Affiliations:** 1 Plant Genome and Development Laboratory (UMR 5096 – CNRS – IRD – UPVD), Institut de Recherche pour le Développement (IRD), Montpellier, France; 2 Agrobiodiversity and Biotechnology Project, International Center for Tropical Agriculture (CIAT), Cali, Colombia; 3 Génoscope, Institut de Génomique, Evry, France; Instituto de Biología Molecular y Celular de Plantas, Spain

## Abstract

A strong postzygotic reproductive barrier separates the recently diverged Asian and African cultivated rice species, *Oryza sativa* and *O. glaberrima*. Recently a model of genetic incompatibilities between three adjacent loci: *S_1_A*, *S_1_* and *S_1_B* (called together the *S_1_* regions) interacting epistatically, was postulated to cause the allelic elimination of female gametes in interspecific hybrids. Two candidate factors for the *S_1_* locus (including a putative F-box gene) were proposed, but candidates for *S_1_A* and *S_1_B* remained undetermined. Here, to better understand the basis of the evolution of regions involved in reproductive isolation, we studied the genic and structural changes accumulated in the *S_1_* regions between orthologous sequences. First, we established an 813 kb genomic sequence in *O. glaberrima*, covering completely the *S_1_A*, *S_1_* and the majority of the *S_1_B* regions, and compared it with the orthologous regions of *O. sativa*. An overall strong structural conservation was observed, with the exception of three isolated regions of disturbed collinearity: (1) a local invasion of transposable elements around a putative F-box gene within *S_1_*, (2) the multiple duplication and subsequent divergence of the same F-box gene within *S_1_A*, (3) an interspecific chromosomal inversion in *S_1_B*, which restricts recombination in our *O. sativa*×*O. glaberrima* crosses. Beside these few structural variations, a uniform conservative pattern of coding sequence divergence was found all along the *S_1_* regions. Hence, the *S_1_* regions have undergone no drastic variation in their recent divergence and evolution between *O. sativa* and *O. glaberrima*, suggesting that a small accumulation of genic changes, following a Bateson-Dobzhansky-Muller (BDM) model, might be involved in the establishment of the sterility barrier. In this context, genetic incompatibilities involving the duplicated F-box genes as putative candidates, and a possible strengthening step involving the chromosomal inversion might participate to the reproductive barrier between Asian and African rice species.

## Introduction

Speciation is one of the central processes in evolution. Geographic isolation of previously interbreeding populations and their subsequent evolutionary divergence appear as one of the mechanisms that influence the emergence of reproductive barriers, and then could promote the creation of new species. In this type of speciation, called geographic speciation or allopatric speciation, reproductive isolation may be achieved by prezygotic or postzygotic barriers (reviewed in [1]). The mechanism that allows the establishment of intrinsic postzygotic reproductive barriers was explained by the Bateson, Dobzhansky and Muller (BDM) model of genetic incompatibilities [2], [3], [4], as an accumulation of genetic substitutions through the divergent evolution of epistatic genes. These substitutions, which could be either adaptive or neutral in the same population, may be deleterious once confronted in the hybrids. The BDM model was recently supported by the identification of incompatibilities between genes acting as postzygotic barriers [5], [6], [7], [8], [9], [10], [11]. In strictly geographic speciation, the complete absence of gene flow between populations suggests a predictable uniform pattern of divergence across genomic regions. By contrast sympatric speciation, that allows a certain level of continuous gene flow between populations, leads to a mosaic genome structure with disparate sequence divergence, as demonstrated in whole genome approaches between closely related animal species [12], [13]. The regions of high divergence are more likely to be associated with the presence of loci that maintain the genetic isolation of species or populations, since they are influenced by the strong diversifying pressure exerted over these loci [14].

Cultivated rice belongs to two distinct species: *Oryza sativa* L. originated from Asia but now cultivated worldwide, and *Oryza glaberrima* Steud originated and restricted to West Africa. Despite the remarkable morphological and agricultural trait differences of Asian and African cultivated rice species [15], their wild relatives diverged recently from a common ancestor, approximately 0.6 to 0.7 million years ago [16], [17], [18]. An Asian origin and ancestral animal dispersal to Africa of *Oryza* species were proposed to explain the biogeographic pattern of African rice [19], [20]. Then, the posterior domestication processes of *O. sativa* in Asia and *O. glaberrima* in Africa took place independently. Despite a complex history, it appears that *japonica* and *indica* subspecies of *O. sativa* were domesticated from each other from pre-differentiated populations of *O. rufipogon* in Asia, approximately 7,000 years ago [21], [22], *O. glaberrima* was domesticated from its wild relative *O. barthii*, in the Niger River delta in Mali approximately 3,000 years ago [23], [24].

*O. sativa* and *O. glaberrima* are reproductively isolated, limiting significantly the use of the genetic potential of *O. glaberrima* for the improvement of Asian rice. Therefore, the identification and characterization of the genetic factors that affect fertility in the interspecific hybrids will allow an easier use of *O. glaberrima* in rice breeding programs, and a better understanding of the nature of postzygotic barriers. Reproductive isolation between *O. sativa* and *O. glaberrima* is mediated by a strong postzygotic barrier, which results from the action of several loci over the fertility of the F_1_ hybrids [25], [26]. Among them, the *S_1_* locus plays a central role. By regular and innovative mapping approaches, the *S_1_* locus was recently fine mapped [27], [28]. Additionally, the existence of two linked epistatic loci was inferred from genetic data, and a model based on a BDM incompatibility between the three adjacent loci (*S_1_A*, *S_1_* and *S_1_B*) was proposed to explain the allelic elimination of female gametes [27]. Finally, a gene coding for a putative F-box protein, and a Pack-Mule carrying a segment of an AP2 homolog were inferred as the most likely candidate factors for *S_1_*.

Here we study patterns of divergence and evolution in the *S_1_A*, *S_1_* and *S_1_B* loci (called here *S_1_* regions), by genomic comparative approaches between orthologous sequences in *O. glaberrima* cv. CG14 and *O. sativa* sp. *japonica* cv. Nipponbare. Our objectives were (1) to establish the genomic sequence of the *S_1_* regions in *O. glaberrima*, (2) to identify patterns of divergence and evolution in the *S_1_* regions, and (3) to determine whether these patterns are informative to better understand the origin and the mechanism of the *S_1_* postzygotic reproductive barrier.

Our results suggest that the *S_1_* regions of *O. sativa* and *O. glaberrima* have undergone no drastic variation in its recent divergence and evolution, implying that the accumulation of small genic changes, following a Bateson-Dobzhansky-Muller (BDM) model, might be the major evolutionary force behind this reproductive barrier. In this context, genetic incompatibilities involving the duplicated F-box genes as putative candidates, and a possible strengthening step involving the chromosomal inversion might participate to the reproductive barrier between Asian and African rice species.

## Results

### Sequencing of the *S_1_*, *S_1_A* and *S_1_B* loci in *O. glaberrima*

In a previous work we described the fine genetic and physical mapping, sequencing, and comparative analysis of the *S_1_* locus [27]. Additionally we detected the presence of two other loci that interact epistatically with *S_1_* to cause the allelic elimination of female gametes produced by the F_1_ hybrids. In order to study these two additional loci, we sequenced the seven remaining clones from the *O. glaberrima* cv. CG14 BAC library [29] that constitute the minimum tiling path (MTP) established around *S_1_* [27]. The eight sequenced clones (including the one from our previous work) were obtained with a coverage ranging between 11 and 14× and an error rate below 1 base per 100 kb. They account altogether for 1,102 kb of sequences which, once assembled, constitute an 813 kb contig, referred here as “the *S_1_* regions”. The seven newly obtained sequences are available with the following EMBL accession numbers: FP340539 (OG-BBa0041E07); FP340540 (OG-BBa0056F23); FP340541 (OG-BBa0088O22); FP340542 (OG-BBa0045G15); FP340544 (OG-BBa0017A24); FP340545 (OG-BBa0066E18); and FP340546 (OG-BBa0093E08).

### Determining the bounds of the *S_1_A* and *S_1_B* loci

**Figure 1:**
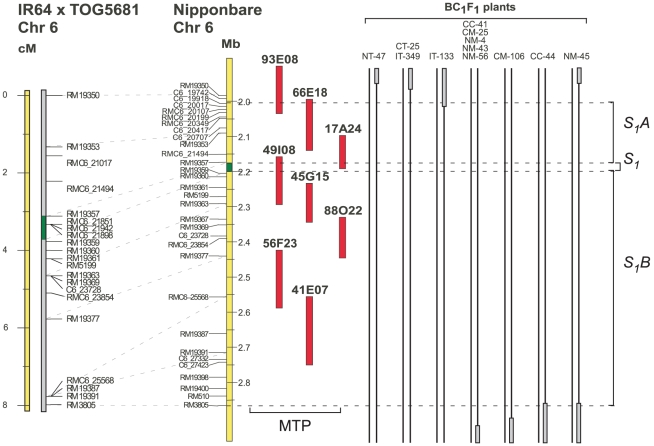
Genetic and physical maps of *S_1_*, *S_1_A* and *S_1_B* loci. Comparison between the genetic and physical maps of the *S_1_* loci, showing the interval of high probability of presence of *S_1_A* and *S_1_B* loci, determined by identifying the recombination breakpoints. The *S_1_A* locus is completely represented in the *O. glaberrima* physical map, while approximately 110 kb are missing from *S_1_B*. A straight line and a gray solid bar represent the *O. sativa* and *O. glaberrima* chromosomes respectively.

In order to find the bounds of the segment that bears the *S_1_A* and *S_1_B* loci, we designed a set of new polymorphic markers, based on the sequence comparison of the orthologous regions between Nipponbare and CG14. Marker evaluation was carried out in the plants that limited the *S_1_A* and *S_1_B* loci [27], in order to find the approximated recombination sites. Using this approach the *S_1_A* locus was reduced to 171 kb, while no reduction was obtained for *S_1_B* locus, remaining as a 654 kb sequence (Figure 1). The 813 kb sequence thus covers completely the *S_1_A* and *S_1_* loci and partially the *S_1_B* locus, since the 102 kb proximal segment from the *S_1_B* locus (markers C6_27332 to RM3805) is not included in the physical map and the sequenced clones. This segment was not found available either in the *O. glaberrima* (cv. GC14) genome project, due to a gap in the obtained physical map (R.A. Wing and P.R. Marri, personal communication). Additionally, microsatellite markers positioned within the gap segment in Nipponbare show an incongruent pattern of amplification and non-amplification in our *O. glaberrima* accessions (Data not shown). These data suggest that the structure of the proximal region of the *S_1_B* segment might be significantly different between *O. sativa* and *O. glaberrima*. Nevertheless, the nature and exact location of these differences remain unknown.

### Sequence annotation and organization of the 813 kb of the CG14 *S_1_* regions

**Figure 2:**
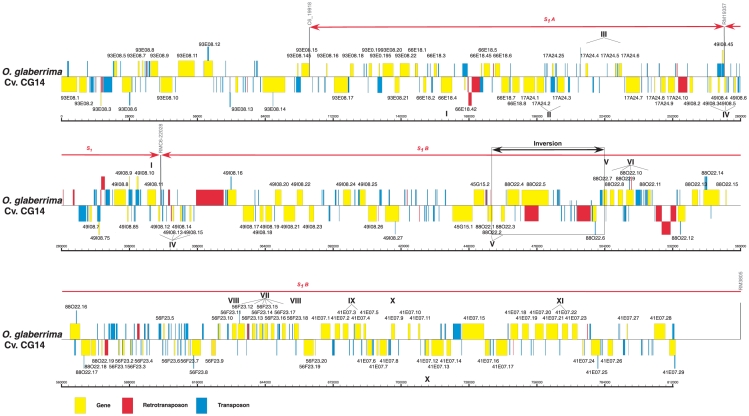
Physical map and annotation of the 813 kb region of the *S_1_*, *S_1_A* and *S_1_B* loci in *O. glaberrima* cv. CG14. Yellow, blue and red boxes represent genes, transposons and retrotransposons, respectively. TEs nested into others TEs or genes are raised above their insertion sites. Markers used in the genetic map are indicated in gray. A black arrow indicates a large sequence inversion, relative to *O. sativa* ssp. *japonica* cv. Nipponbare. Regions spanning the *S_1_*, *S_1_A* and *S_1_B* loci are indicated. Roman numerals indicate duplicated gene families listed in Table 1.

The sequences of the CG14 BAC clones were annotated in detail. Gene and transposable element (TE) annotations are indicated in Figure 2. In total, 143 non-TE related coding regions were annotated, which corresponds to a gene density of about one gene per 6 kb of genomic sequence (Table S1). Most of predicted genes were confirmed by identification of protein domains, BLASTX homologies in Swiss-Prot database or BLASTN homologies with nucleotide databases. On the 143 predicted genes, 96.3% showed strong BLASTN homology with *O. sativa* genomic and full-length cDNA sequences. Seven pseudogenes were identified by the presence of fragmented coding regions lacking start codons, or by the presence of stop codons in the frame of exons. Of the 143 predicted genes, 35 (25%) belong to eleven distinct duplicated gene families scattered along the 813 kb analyzed, coding for F-box (gene families I and VII), LRR proteins (II), putative homeobox (III), Early nodulin-like (IV), Pectate lyase (V), Transferase (VI and X), Esterase/Lipase (VIII), Cystein synthase (IX) and Methylase proteins (XI) (Table 1 and Figure 2). Copy numbers of duplicated genes vary from two to seven genes. Most of the duplicated gene families were organized in clusters of relatively adjacent duplicated genes, with the exception of the two genes from family I (F-box family) separated by 154 kb of genomic sequence. The majority of the duplicated gene families were found to be arrayed in tandem while only three families showed duplicated genes in opposite orientations (I, V and X, Table 1). As illustrated by the *S_1_* regions, the rice genome appears to be shaped by a relatively high number of local gene duplications [30].

**Table 1:** List of identified gene families in the *O. glaberrima S_1_* regions.

Duplicated gene family	Gene Name	Putative *O. sativa* (Nipponbare) orthologous gene	Putative Function	Position in the CG14 contig (bp)	Orientation
**I**	**OG-BBa0066E18.4**	LOC_Os06g04690	Putative F-box protein	156499–161044	−
	**OG-BBa0049I08.11**	LOC_Os06g04980	Putative F-box protein	315435–318859	+
**II**	**OG-BBa0017A24.2**	LOC_Os06g04830	Putative LRR protein	195957–198903	−
	**OG-BBa0017A24.3**	LOC_Os06g04840	Putative LRR protein	204593–207258	−
**III**	**OG-BBa0017A24.4**	LOC_Os06g04850	Putative protein	216110–217001	+
	**OG-BBa0017A24.6**	LOC_Os06g04870	Putative protein	229920–230963	+
**IV**	**OG-BBa0049I08.3**	LOC_Os06g04930	Putative ENOD93 protein	267102–267855	−
	**OG-BBa0049I08.5**	LOC_Os06g04940	Putative ENOD93 protein	273413–274012	−
	**OG-BBa0049I08.6**	LOC_Os06g04950	Putative ENOD93 protein	277419–277981	−
	**OG-BBa0049I08.12**	LOC_Os06g04990	Putative ENOD93 protein	320002–320651	−
	**OG-BBa0049I08.13**	LOC_Os06g05000	Putative ENOD93 protein	325881–326408	−
	**OG-BBa0049I08.14**	LOC_Os06g05010	Putative ENOD93 protein	328903–329477	−
	**OG-BBa0049I08.15**	LOC_Os06g05020	Putative ENOD93 protein	332839–333419	−
**V**	**OG-BBa0088O22.2**	LOC_Os06g05209	Putative Pectate lyase protein	456489–457642	−
	**OG-BBa0088O22.3**	LOC_Os06g05260	Putative Pectate lyase protein	461242–462685	−
	**OG-BBa0088O22.7**	LOC_Os06g05272	Putative Pectate lyase protein	503408–504867	+
**VI**	**OG-BBa0088O22.8**	LOC_Os06g05284	Putative Transferase	506199–507791	+
	**OG-BBa0088O22.9**	LOC_Os06g05300	Putative Transferase	509461–511398	+
	**OG-BBa0088O22.10**	LOC_Os06g05310	Putative Transferase	512912–515793	+
	**OG-BBa0088O22.11**	LOC_Os06g05320	Putative Transferase	521812–522540	+
**VII**	**OG-BBa0056F23.12**	LOC_Os06g05560	Putative protein	632773–635500	+
	**OG-BBa0056F23.13**	LOC_Os06g05580	Putative F-box protein	638676–639857	+
	**OG-BBa0056F23.14** *	LOC_Os06g05590	Putative F-box protein	641606–642834	+
	**OG-BBa0056F23.15**	LOC_Os06g05600	Putative F-box protein	643777–644970	+
	**OG-BBa0056F23.16**	LOC_Os06g05610	Putative F-box protein	646377–647636	+
	**OG-BBa0056F23.17** *	LOC_Os06g05620	Putative F-box protein	650971–652272	+
**VIII**	**OG-BBa0056F23.11**	LOC_Os06g05550	GDSL esterase/lipase protein	630402–632063	+
	**OG-BBa0056F23.18**	LOC_Os06g05630	GDSL esterase/lipase protein	655875–657747	+
**IX**	**OG-BBa0041E07.3**	LOC_Os06g05690	putative Cystein synthase protein	676063–678199	+
	**OG-BBa0041E07.4**	LOC_Os06g05700	putative Cystein synthase protein	681007–683334	+
**X**	**OG-BBa0041E07.9**	LOC_Os06g05750	Putative Transferase protein	695968–697392	+
	**OG-BBa0041E07.12**	LOC_Os06g05790	Putative Transferase protein	710049–711485	−
**XI**	**OG-BBa0041E07.21**	LOC_Os06g05900	Putative Methylase protein	759933–764709	+
	**OG-BBa0041E07.22**	LOC_Os06g05910	Putative Methyltransferases	766462–768723	+

*Pseudogene.

In total 380 known TE were identified representing 18.3% of the genomic sequence. Of the 380 elements, 37 were classified as class I retroelements (Table S2). Interestingly, ten annotated transposons overlaped with predicted coding regions. These transposons were classified as pack-MULE since they showed similarities with Mutator-like elements and contained embedded coding sequences [31]. Among enclosed coding regions, six were classified as pseudogene due to the presence of frame-shift mutations or the complete absence of start codons. Only three pack-MULE displayed similarities to proteins with known functions (Table S3).

### Sequence comparisons of orthologous *S_1_* regions

**Figure 3:**
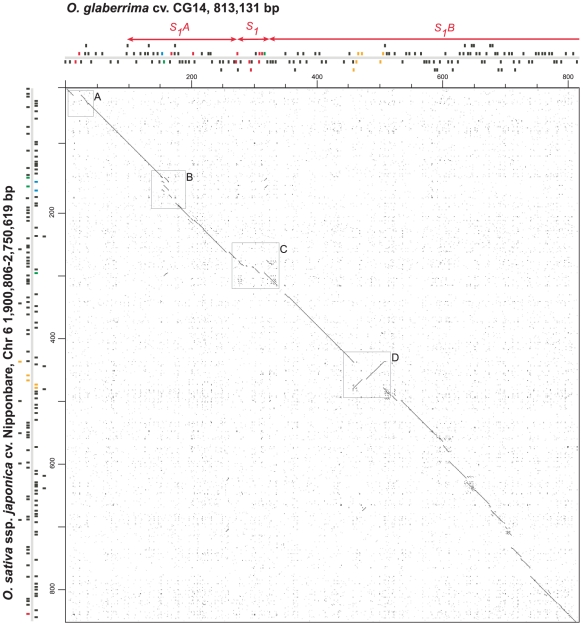
Orthologous sequence comparisons between the 813 kb and 847 kb from the *O. glaberrima* cv. GC14 and *O. sativa* ssp. *japonica* cv. Nipponbare *S_1_* regions. Comparisons were performed using dot plot alignment of the CG14 sequence (horizontal axis) against the Nipponbare sequence (vertical axis; coordinates 1,900,806–2,750,619-bp on chromosome 6). The *S_1_A*, *S_1_* and *S_1_B* regions are indicated along the horizontal axis. Positions and orientation of genes are symbolized by black and colored boxes along X and Y-axes. Colored boxes represent genes that disrupted microcollinearity. Clear boxes in the dot blot underline four large regions showing a strong disruption in the microcollinearity.

The orthologous regions were identified in the *O. sativa* ssp. *japonica* cv. Nipponbare public sequence as a stretch of 847 kb between coordinates 1,900,806 and 2,748,457 on chromosome 6. The orthologous regions in *O. sativa* ssp. *indica* cv 93-11 sequence was also used for comparative analysis and consist of a stretch of 1,077 kb broken by 152 ambiguous segments (with ‘N’), representing gaps, that sometimes did not allow accurate comparative analysis. Pairwise comparisons between CG14 and Nipponbare *S_1_* regions revealed the presence of stretches of highly conserved segments interrupted by a limited number of zones with significant alterations. Most of the sequence variations involved mechanisms of sequence insertions, deletions, duplications, and a large sequence inversion, as illustrated in Figure 3. Sequence variation was not limited to intergenic regions since they overlap with different segments that include coding genes. Detailed analysis of the collinearity was then performed between the non-TE genes located within the CG14 and Nipponbare orthologous regions. All CG14 predicted genes were used as queries to BLAST them against Nipponbare genes within the *S_1_* regions, to generate a matrix of distance between genes used to draw the relationships between orthologous and paralogous genes (Figure S1). Most of the genes (>90%) were found conserved in the same order and orientation between the two orthologous sequence. Seventeen and 11 predicted genes, respectively in CG14 and Nipponbare, were found to be involved in mechanisms that disrupt the collinearity. First, eleven genes were present in CG14 and absent in Nipponbare, while only three extra genes were found in the Nipponbare segment. Of the eleven extra genes predicted in CG14, seven were enclosed into pack-MULE elements (66E18.45, 17A24.25, 49I08.4, 49I08.45, 49I08.75, 49I08.9 and 49I08.10, Table S1 and S3). In Nipponbare, two of the three extra genes (Os06g04710 and Os06g05470) were classified as expressed proteins. This result suggests that a significant number of collinearity disruptions in the *S_1_* regions may be produced by gene movement mechanisms, such as the transposition of pack-MULE elements. These disruptions were mainly distributed into three large segments along the *S_1_* regions and one region outside the *S_1_* regions (Figure 3). Detailed comparisons between CG14 and the two sub-species of *O. sativa* (ssp. *japonica* cv. Nipponbare and ssp. *indica* cv. 93-11) were carried out in these sites, in order to investigate the molecular mechanisms involved.

Around the 93E18.3 and 93E18.5 CG14 genes, located in the flanking region of the genetic interval of *S_1_A*, sequence comparisons showed a large insertion of 13,123 bp of sequence in CG14 relative to Nipponbare (Figure 3, box A). This extra segment carries a predicted pseudogene (93E18.3) and a gene coding for a putative protein (93E18.5), absent in *O. sativa* (Nipponbare and 93-11; data not shown). It was not possible to identify the mechanism that originated this insertion (or deletion in Nipponbare), since its extremities have no similarity with known TEs, and no traces of short duplications were clearly visible at the insertion site in CG14. Although the insertion (or deletion) of large segments containing genes appears to be common in rice compared to distant species such as *Brachypodium* [32], such rearrangement hasn't been previously reported between closely related rice species.

**Figure 4:**
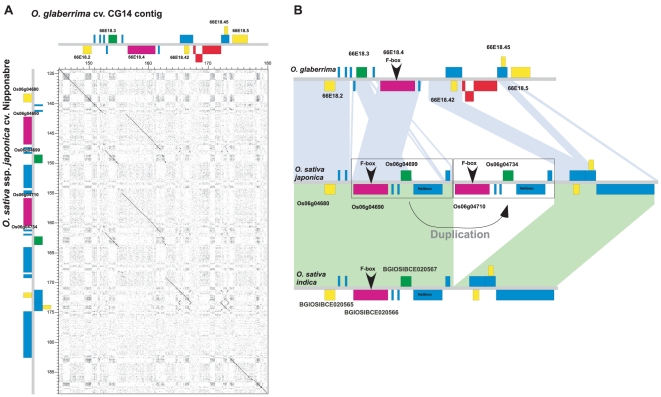
Comparisons of the orthologous sequences around the 66E18.3/66E18.4 CG14 genes. **A**. Dot plot comparison between the genomic region of genes 66E18.3 and 66E18.4 from CG14 (horizontal axis; coordinates 145–180 kb) against the *O. sativa* ssp. japonica cv. Nipponbare orthologous sequence (vertical axis; coordinates 2,034,806–2,088,806-bp on chromosome 6). **B**. Schematic representation of the comparison between the genomic region of genes 66E18.3 and 66E18.4 and their orthologous genes in *O. sativa* ssp. *japonica* cv. Nipponbare and *O. sativa* ssp. *indica* cv. 93-11. Colored backgrounds link orthologous regions. Boxes symbolize the positions of transposons and helitrons elements (light blue), Retrotransposons (red), Putative protein gene 66E18.3 and its orthologs (green), Putative F-box protein gene 66E18.4 and its orthologs (purple), and other genes (yellow).

Comparisons between orthologous sequences around 66E18.3 (Putative protein) and 66E18.4 (Putative F-box protein) genes indicated that both CG14 and Nipponbare regions have undergone a multitude of small changes since the loci have diverged from a common ancestor (Figure 3, box B). Here collinearity is altered by a local gene order alteration involving both genes, compared to the positions of the orthologous genes from *O. sativa* (ssp. *japonica* and *indica*) (Figure 4). In addition to the order rearrangement, a several TE insertions (two MITEs and two retrotransposons in CG14, and a large helitron and a transposon in Nipponbare and 93-11) were detected. Furthermore, a block of approximately 13 kb comprising genes Os06g04690, Os06g04699 and one helitron was found duplicated in tandem orientation in Nipponbare but not in 93-11 (Figure 4 B). The mechanisms at the origin of the gene rearrangement between 66E18.3 and 66E18.4 remain unidentified, thus no evolutionary model could be developed. However it seems clear that numerous TEs have been inserted up- and downstream the orthologous genes after the divergence between *O. glaberrima* and *O. sativa*; and that after the divergence between *indica* and *japonica* subspecies, a large duplication occurred in Nipponbare relative to 93-11. Furthermore, comparisons between the F-box duplicated genes in Nipponbare (Os06g04690 and Os06g04710) reveal significant changes. Due to frame-shift mutations, the predicted Os06g04710 gene is shorter than the duplicated Os06g04690 gene, resulting in a predicted protein that lacks the N-terminal region of the F-box domain. Altogether, these events indicate that this region may represent an intense spot of recent divergence between *O. glaberrima* and *O. sativa*, but also within *O. sativa* subspecies.

At the 49I08.7/49I08.11 genes region (Figure 3, box C), localized within the *S_1_* locus, the collinearity was altered by the presence of a total of five extra genes in *O. glaberrima* compared to *O. sativa* [27]. Most of them (49I08.75; 49I08.9 and 49I08.10) appear to be enclosed within pack-MULE elements, suggesting that massive re-localization of these elements in the *O. glaberrima* region may be here the mechanism for collinearity perturbation (Table S3).

**Figure 5:**
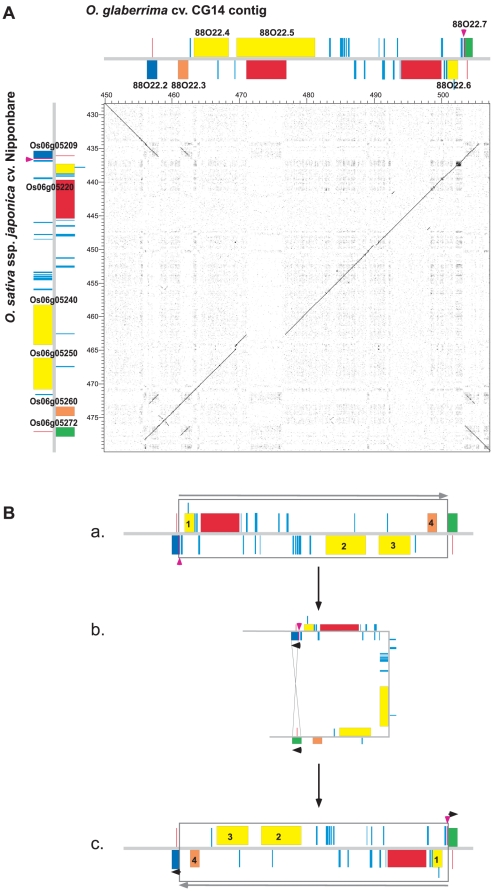
Comparisons of the orthologous sequences around the 88O22.2/88O22.7 CG14 *g*enes. **A**. Dot plot comparison and structures between the genomic region of genes 88O22.3/88O22.7 from CG14 (horizontal axis; coordinates 445–506 kb) and their orthologous in Nipponbare (vertical axis; coordinates 2,336,995–2,376,853-bp on chromosome 6). **B**. A model for the generation of the chromosomal inversion between *O. glaberrima* and *O. sativa*. **a**. Hypothetical ancestral segment (identical to the organization in the Nipponbare genome); **b**. Breakpoints occur by homologous recombination between two homologous genes coding for Pectate lyase proteins. **c**. Segment in *O. glaberrima*. Boxes symbolize the positions of Pectate lyase duplicated genes (blue, green and orange), transposons elements (light blue), Retrotransposons (red), and other genes (yellow). Purple boxes and arrowheads indicate the positions of twelve extra amino acids between duplicated Pectate lyase genes.

Dot plot alignment of the CG14 and Nipponbare orthologous sequences around genes 88O22.2/88O22.7 evidenced a paracentric chromosomal inversion of approximately 45 kb (Figure 3, box D). This inversion involved four different coding genes (88O22.3, 88O22.4, 88O22.5 and 88O22.6) in CG14, perturbing gene orders and orientations. A detailed comparison between orthologous sequences was carried out in order to identify the chromosomal inversion breakpoints and to investigate the process responsible of such rearrangement. Close analysis indicated that the distal and proximal inversion breakpoints contain gene duplications in both species (respectively 88O22.2/88O22.7 genes and Os06g05209/Os06g05272 genes). These genes, coding for Pectate lyase proteins, belonged to a locally duplicated gene family composed of three gene copies (Family V, Table 1). Duplicated genes at the edge of the inversion were nearly identical, with the exception of the first 36 extra-nucleotides at the 5′ end of 88O22.7 and Os06g05209 genes, resulting in twelve extra amino-acids for each gene (purple boxes and arrowheads, Figure 5 A). All genes located in the inversion breakpoints in CG14 and Nipponbare appear intact and seem putatively functional (even after switching their upstream segment), since their coding regions are identical. A tentative model for the chromosomal inversion process is depicted in Figure 5 B. In the ancestral fragment—here the structure of the fragment is identical to the Nipponbare one—, two homologous Pectate lyase genes in opposite orientations flanked an internal region of 45 kb (Blue and green boxes, Figure 5 B). A mechanism of homologous recombination between inverted Pectate lyase genes occurred, leading to an inversion of the internal region and the exchange of upstream regions of Pectate lyase genes.

Unfortunately the reduced quality of the sequence assembly for 93-11 did not allow us to confirm the presence of the chromosomal inversion between *O. glaberrima* and *O. sativa ssp. indica*. Nevertheless, a mapping analysis based on BLAST alignments of available BAC end sequence (BES) pairs from seven different *Oryza* species [33] around and within the inversion breakpoints, clearly indicated that the inversion structure is identical between *O. glaberrima* and four other *Oryza* species (*O. nivara*, *O. officinalis*, *O. alta* and *O. australiensis*). On the contrary, the mapping of BESs from *O. sativa* (ssp. *japonica* cv Nipponbare) and *O. rufipogon*, the wild ancestor of *O. sativa*, suggest a different genomic structure compared to the *O. glaberrima* region (Figure S2).

Since chromosomal inversions are known to suppress genetic recombination between normal and inverted chromosomal segments, we evaluated the recombination rates in our *O. sativa*×*O. glaberrima* backcross populations [27] around and within the inversion. The genetic map obtained from 779 BC_1_F_1_ plants, after a high marker saturation in the site of the structural variation, showed a complete absence of genetic recombination between the inversion breakpoints, in contrast to the recombination rates found in the rest of the 813 kb contig (Figure S3). These data suggest that the chromosomal inversion represents an inter-specific rearrangement between *O. glaberrima* and *O. sativa*, which strictly restricts recombination within its limits. To our knowledge, this is the first report of a chromosomal inversion initiated by duplicated genes in plants. The considerable number of locally duplicated genes in rice may offer potential recombination targets for chromosomal rearrangements mechanisms involving coding regions [30].

### Transposable elements participated to the dynamic evolution of *S_1_* regions

Beside the alteration of the order and orientation of genes through re-localization of pack-MULE elements, comparative analysis between the Nipponbare and CG14 *S_1_* regions reveals changes of the genomic structure due to differential insertion or deletion of a multitude of transposable elements. Since the divergence of the two species, more than 117 kb of TE (13% of the segment) were inserted in Nipponbare, against 72 kb (9%) in CG14. The size difference observed at the *S_1_* regions, mainly due to the insertion of long full-length LTR retrotransposons, is in agreement with the genome size difference between *O. sativa* (434 Mb) and *O. glaberrima* (352 Mb) [34]. Most of the TEs appear randomly inserted along the *S_1_* regions, with the apparent exception of the TE accumulation that occurred in the *S_1_* locus (Figure 3). Here, the CG14 segment has undergone a 1.5× sequence size increase due to the local accumulation of TEs in the neighborhood of the *S_1_* candidate gene (49I08.11) [27]. Beside the *S_1_* locus, successive but isolated TE insertions responsible for the observed interruptions on the collinearity occurred specifically in Nipponbare, downstream the chromosomal inversion (Figure 3).

### Gene divergence in *S_1_* regions between *O. sativa* and *O. glaberrima*

**Figure 6:**
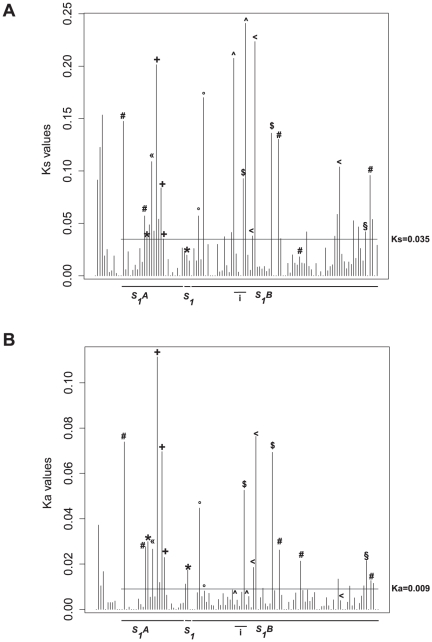
Representation of Ka and Ks values between 109 *O. sativa* and *O. glaberrima* orthologous genes in the *S_1_* regions. **A**. Representation of Ks values. The horizontal line is the mean value of Ks (Ks = 0.035) for the 109 analyzed orthologous genes in the *S_1_* regions. **B**. Representation of Ka values. The horizontal line is the mean value of Ka (Ka = 0.09) for the 109 analyzed orthologous genes in the *S_1_* regions. Lines below graphics represent analyzed genes located into *S_1_A*, *S_1_*, *S_1_B* loci, and those within the chromosomal inversion (i). Symbols represent different divergent genes with similar annotated function or unknown function as follows in *O. glaberrima*: (*) 66E18.4 and 49I08.11 (F-box proteins), (“) 66E18.6 (HAD phosphatase protein), (+) 66E18.8, 17A24.2 and 17A24.3 (LRR proteins), (°) 49I08.16 and 49I08.18 (Putative Serine/threonine protein kinases), (∧) 88O22.2 and 88O22.7 (Pectate lyase located at breakpoint inversion), (§) 41E07.23 (PRR protein) and (<) 88O22.10, 88O22.11 and 41E07.12 (Putative transferase proteins), ($) 88O22.6 and 88O22.19 (Hypothetical proteins) and (#) 93E08.17, 66E18.3, 56F23.3, 56F23.12 and 41E07.25 (Putative proteins).

Of the 143 annotated genes in the sequenced regions, 120 were used for pairwise comparisons and divergence analysis with their respective Nipponbare orthologous genes, from which 109 fell into the *S_1_* regions. Twenty-three CG14 genes were not analyzed due to the absence of a Nipponbare orthologous gene, deep annotated gene structure differences between them, or because one of the two was annotated as a pseudogene. Similar analyses were carried out as a control, using two other published genomic regions in CG14 where no reproductive isolation region between the two species has been previously reported: (1) the *ADH* region on the short arm of chromosome 11, containing 13 annotated genes [18], and (2) the *MOC1* region on the long arm of chromosome 6, containing 17 annotated genes [35]. The mean rate of non-synonymous substitutions (Ka) and synonymous substitutions (Ks) across the *S_1_* regions are respectively 0.009 and 0.035 (Figure 6). No statistical difference in the mean levels of Ka and Ks (respectively P = 0.771 and P = 0.317) was found when comparing with the *ADH* (Ka = 0.007, Ks = 0.040) and *MOC1* (Ka = 0.004, Ks = 0.020) regions (Table S4 and Figure S4), suggesting that the *S_1_* as well as the *ADH* and *MOC1* regions are globally under identical evolution rates of protein-coding genes. Despite an overall uniformity of Ka and Ks values along the *S_1_* regions, several isolated peak values were higher than both background and mean values (Figure 6). Functions and Gene Ontology (GO) of annotated genes harboring increased Ks and/or Ka values were investigated. Of the 21 genes showing elevated Ks or Ka (at least two times the Ks or Ka mean values, indicated by symbols on Figure 6), 14 have known functions and are classified into the following categories of the “biological process” of Gene Ontology: response to biotic stimulus, protein modification, signal transduction and response to endogenous stimulus; and into the “molecular function” categories: kinase activity, nucleotide binding, protein binding, transferase activity, catalytic activity and hydrolase activity. The function and ontology of these genes suggest that high divergence may be a consequence of a local and accelerated evolution possibly driven by adaptation [36] (Figure 6). The Ka/Ks ratio was also calculated to characterize the evolution of protein-coding sequences in the *S_1_* loci (Table S5). More than 85% of the genes were found to be under strong purifying selection, six genes to have a neutral evolution, while the 11 genes with the highest Ka/Ks values seemed to be evolving under positive selection or relaxed selective constraint. Finally, we investigated whether high gene divergence is globally associated to structural variations such as transposable element abundance, duplications and chromosomal inversions. No clear association was found between divergent genes and transposable element abundance as illustrated by the *S_1_* locus, where a clear accumulation of TEs was observed in CG14 compared to Nipponbare [27], with no significant effect on gene divergence. Similarly, within the chromosomal inversion, no peak of high divergence was associated to genes at the relative exception of the hypothetical gene 88O22.6. The high Ks values observed for the duplicated Pectate lyase genes might be generated by mosaic gene structures since these genes are located at the inversion breakpoints (Figure 6 A).

### Characterization of duplicated F-box genes in the *S_1_* regions

The gene 49I08.11 coding for a putative F-box protein has been proposed as a putative candidate gene for the *S_1_* locus, on the basis of the protein function of homologous genes and its high degree of divergence between the two species [27]. Sequencing of the 813 kb of the *S_1_* regions in *O. glaberrima* revealed a duplicated copy of the 49I08.11 F-box gene, located 154 kb apart in the *S_1_A* locus (gene 66E18.4) (Figure 2 and Table S1). The duplicated genes exhibited high overall sequence similarities (90.7% of nucleotide identities). Both *O. glaberrima* F-box genes were found conserved in the orthologous *O. sativa* region. Altogether these data suggest that the duplicated F-box 66E18.4 gene may also be a valuable candidate gene for *S_1_A*.

To study evolution of this F-box gene family, detailed gene comparisons were performed at the *S_1_A* and *S_1_* loci. In the *S_1_* locus, nucleotide and amino acid alignments showed significant sequence variations between orthologous F-box genes in *O. glaberrima* and *O. sativa* (Figure S5). The elevated Ka and Ks values and the calculated Ka/Ks ratio (Table S5) suggest an accelerated but neutral evolution, while the majority of genes in the *S_1_* regions appears to be under a purifying evolution. Besides coding region evolution, the CG14 49I08.11 F-box gene is embedded in an accumulation of TEs that reshaped its upstream and downstream regions. Furthermore its gene structure also evolved through the insertion of a non-autonomous transposon nested into the fourth intron of the gene [27]. In the *S_1_A* locus, a unique F-box gene is present in CG14 (66E18.4) and in *O. sativa* ssp. *indica* (BGIOSIBCE020566), compared to two tandemly duplicated orthologous genes in *O. sativa ssp. japonica* (Os06g04690 and Os06g04710). While the structure of the Os06g04690, BGIOSIBCE020566 and 66E18.4 genes appears to be the same, the 5′ part of the Os06g04710 gene exhibits several frame-shifts, resulting in a shorter predicted protein that lacks the N-terminal region of the F-box domain. Beside this variation, the alignments between the *S_1_A* F-box genes showed numerous polymorphisms at the amino-acid levels (Figure S5), which shaped the corresponding phylogenetic tree (Figure S6). Ka/Ks rates, calculated for these coding regions, suggest a similar evolution to the one observed for the *S_1_* F-box genes. Together these results suggest an accelerated evolution of these F-box genes that drives the divergence of the *O. sativa* and *O. glaberrima* orthologous genes, but also between the two *O. sativa* subspecies. These evidences allow considering these duplicated genes as potential candidates for the *S_1_A* and *S_1_* loci.

## Discussion

The growing availability of whole genome sequences and comparative analysis of gene divergence have helped evolutionists to deduce the presence of reproductive barriers within highly divergent genomic regions [14], and even to infer the possible path of speciation for several related species [12], [13], [37]. Whole genome sequence comparisons between *indica* and *japonica* subspecies of Asian rice have also led to the identification of large regions of high polymorphisms, whose origins have been associated with geographical differentiation, reproductive barriers, subsequent independent domestications, and a more recent admixture possibly mediated by human migration [36]. However no direct comparison between experimentally validated postzygotic isolating loci has been performed so far at the sequence level, to directly investigate in detail the genomic evolution of such regions.

Between the two cultivated rice species, the *S_1_* locus acts as the strongest postzygotic reproductive barrier, having an important role on their origin and conservation. In a previous work, we described the fine genetic and physical mapping of the *S_1_* locus. Additionally we detected the presence of two other loci (*S_1_A* and *S_1_B*) that interact epistatically with *S_1_* to cause the allelic elimination of female gametes produced by the F_1_ hybrids [27]. Based on available data, we build a genetic model where BDM incompatibilities between the alleles of the *O. sativa* and *O. glaberrima S_1_A*, *S_1_* and *S_1_B* loci are provoking the female gamete elimination and the strong transmission ratio distortion observed in the hybrids [27]. Our genetic model states that the final allelic frequencies and final survival rates of female gametes are associated to the recombination ratio between the three epistatic loci, their segregation during meiosis, and the alleles (*indica* or *japonica*) confronted in a given cross. In order to understand the basis of the evolution of the *S_1_* genomic regions, and to infer possible gene candidates or mechanisms behind this reproductive barrier, we sequenced the seven remaining *O. glaberrima* clones that constitute the physical map of the *S_1_* regions, and compared them with the orthologous regions in *O. sativa*.

The comparisons revealed that the *S_1_* regions in both species are strongly conserved in terms of genomic structure and coding sequence divergence. Three isolated regions showing a disturbed collinearity were identified concerning: (1) local invasion of transposable elements (mainly Pack-MULEs carrying remnant of coding genes) around a putative F-box gene, candidate gene for the locus *S_1_*, (2) multiple duplication and subsequent divergence of the same F-box gene, within *S_1_A*, (3) and an interspecific chromosomal inversion in *S_1_B*. Additionally, we showed that most of the genes in the *S_1_* regions undergone a strong purifying selection, with the exception of few isolated divergent genes. These genes belong to functional categories known to confer adaptive advantages, and their highly divergent evolution could be a consequence of local adaptation to the African or Asian environments, or of human selection following the independent domestication processes. The pattern of evolution of a genomic region involved in a reproductive barrier could provide indications on its establishment, specifically, if it occurred under either an active or a restricted gene flow [14]. The similar rate of gene divergence between the *S_1_* regions and two other genomic sites not involved in reproductive isolation may suggest a limited gene flow between populations during the establishment of the *S_1_* barrier. In consequence, the geographic localizations of *O. rufipogon* and *O. sativa* in Asia and of *O. barthii* and *O. glaberrima* in West Africa, together with a restricted gene flow could imply that this speciation process is the result of geographical isolation, in agreement with the current hypothesis of a common Asian origin and ancestral migrations to Africa [19], [20]. However a precise estimation of gene flow rate is required to test this hypothesis.

Under this highly conservative context, the *S_1_* barrier between *O. sativa* and *O. glaberrima* appears to have evolved from the divergent evolution of punctual genes and not from large genomic structural rearrangements, as predicted by the BDM model of incompatibilities. A detailed analysis of genes known to be implicated in BDM incompatibilities could help to identify possible candidates for the *S_1_* locus. Recently several molecular studies in animals and plants (including rice) revealed that gene duplication and divergence could be directly involved in postzygotic reproductive barriers concerning BDM incompatibilities in hybrids [9], [10], [11], [38]. In our previous work, two putative candidate genes for the locus *S_1_* were identified: an F-box gene and a Pack-MULE transposon carrying a fragment of a AP2 gene [27]. Interestingly, a strongly conserved copy of the F-box gene from the *S_1_* locus is located in *S_1_A*, constituting the only gene family to be present at two different loci along the *S_1_* regions. The presence of these duplicated genes appears to match well the evolutionary model of an ancestral duplication followed by a divergent evolution of the alleles in each population. In terms of divergence, the F-box genes 66E18.4 and 49I08.11 in *O. glaberrima* cv. CG14 and their respective orthologous genes Os06g04690 and Os06g04710, and Os06g04980 in *O. sativa* cv. Nipponbare, exhibit a significant accelerated but neutral evolution (Table S5), in contrast to the purifying evolution of the majority of genes along the *S_1_* regions. Additionally, the up- and downstream regions of these F-box genes have undergone a multitude of structural variations since *O. sativa* and *O. glaberrima* diverged (including a second gene duplication and divergence in the *S_1_A* locus, in the *japonica* genome), suggesting that a dynamic evolution may be associated to them.

In rice, the implication of F-box proteins in postzygotic barriers has already been reported for the *Sa* intersubspecific male sterility locus. In this case, the selective abortion of microspores is caused by the interaction between the *indica* and *japonica* alleles of a SUMO E3 ligase (*SaM*) and a F-box gene (*SaF*) [7]. This constitutes another argument for considering the hypothesis that the duplicated F-box genes are involved in the sterility barrier mediated by the *S_1_* locus. Even more, the second gene duplication and divergence in the *S_1_A* locus would allow to explain not only the observed differences in the TRD levels found between the *O. glaberrima*×*O. sativa* ssp. *indica* and the *O. glaberrima*×*O. sativa* ssp. *japonica* hybrids [27], but also the presence of the intersubspecific sterility locus *S_10_*, localized on the same genetic position [16], [39]. F-box proteins constitute one of the largest multi-gene families with more than 700 putative genes and pseudogenes in rice [40], [41], [42]. F-box proteins and their SCF (Skp1-Cullin-F-box) complexes are known to be involved in regulatory functions on several processes, such as the progression throughout the meiotic [43], [44] and mitotic divisions [45] during gametogenesis. In our genetic model for the female sterility caused by *S_1_*, only the cells that inherit a compatible allelic combination are able to pursue their development after each cellular division, to form a functional embryo sac [27]. Taking into account the recognized role of F-box proteins in the cell cycle progression, a BDM incompatibility after a hypothetical divergent subfunctionalization or neofunctionalization involving these genes could thus explain the arrested development of some allelic forms of hybrid gametes. Remarkably, the results from a previous F-box protein microarray analysis during rice panicle development evidenced the expression of genes Os06g04690, Os06g04710 and Os06g04980 (probe Os.3577.1.S1_x_at) in whole panicles throughout the meiotic and young microspore stages, and their down-regulation in mutants for the gene *Udh1*, an important transcription factor for meiocyte differentiation [40]. These expression data demonstrate that these genes are expressed at the time and in the tissues where the BDM incompatibility is supposed to take place in the hybrids according to our genetic model. Taking into account the ability of F-box genes to closely interact with other proteins, their evolutionary plasticity, their known role in cell cycle progression and reproductive barriers, and their expression in reproductive tissues, the duplicated copies of the F-box gene appear as the best candidate factors for the *S_1_A* and *S_1_* loci.

The comparative analysis of gene divergence have helped us to identify two genes possibly involved in the sterility barrier caused by the *S_1_A* and the *S_1_* loci, however no plausible candidate was determined for *S_1_B*, since the available sequence only partially spans the locus, and the structure of its proximal region seems to have a different configuration between the two species. However a striking alteration of the collinearity was observed within the *S_1_B* locus, in the form of a 45 kb chromosomal paracentric inversion between CG14 and Nipponbare. Mapping of BES pairs from seven *Oryza* species suggest a similar structure of the inversion region between *O. glaberrima* and both closely and distantly related species; while a different structure was found in *O. sativa* cv. Nipponbare and *O. rufipogon*. These data suggest that the inversion may have occurred recently in *O. rufipogon*, and has been inherited by *O. sativa* after domestication. In addition to the direct genomic sequence comparison between *O. sativa* and *O. glaberrima*, the genetic analysis showed a complete restriction of recombination between markers spanning the inversion in our interspecific BC_1_F_1_ populations. Besides reducing dramatically recombination between inverted and standard non-inverted chromosomes, inversions appear to play a major role in evolution of species [46], [47]. Between the close relatives sympatric species *Drosophila pseudoobscura* and *D. persimilis*, inversions were found within regions associated with hybrid sterility, suggesting that they might have contributed to their speciation process [48]. Moreover, gene divergence was found higher within inverted regions than in non-inverted regions suggesting the occurrence of gene flow between the two species [13]. In contrast to the *Drosophila* example, the genic divergence outside and within the inversion in the rice *S_1_B* locus appears to be quite uniform (Table S5), suggesting that the inversion might have occurred after speciation or at least after the complete geographical isolation of the species. With the chromosomal inversion fixed in the *O. rufipogon*-*O. sativa* species group, its role in the reproductive isolation mechanisms would be limited to an increase of the genetic linkage between the loci involved in this sterility barrier. Since recombination between the three loci plays a key role in the final allelic frequencies and survival rates of female gametes produced by the hybrids [27], it is probable that the restriction of the recombination caused by the inversion would have a strengthening effect over the barrier [49].

The effect of the inversion on the recombination is not the only sign that the *S_1_* barrier could have been strengthened over time. *S_1_* has been described as a complex locus, having different effects over male and female fertility of the *O. sativa* and *O. glaberrima* hybrids. Plants that carry only the *S_1_* locus in a heterozygote state are partially male sterile [25], [28], while heterozygocity at the *S_1_A*, *S_1_* and *S_1_B* loci is necessary to observe partial female sterility [27]. This differential effect over male and female fertility could mean that the barrier has been strengthened over time by sequential accumulations of incompatibilities. Furthermore, the presence of an additional locus (*S_1_C*) in one of the four interspecific populations examined, which has a supplementary deleterious effect over female gamete elimination [27], seems to indicate that an auxiliary strengthening step may be currently under fixation.

### Conclusions

In this work, we have studied the structural and genic divergence of the *S_1_* regions between *O. sativa* and *O. glaberrima*, as a method to understand the basis of their evolution and to infer possible gene candidates or mechanisms working behind this reproductive barrier. The comparisons showed that the *S_1_* regions have undergone no drastic variation in their recent divergence and evolution, suggesting that a small accumulation of genic changes, following a Bateson-Dobzhansky-Muller (BDM) model, might be involved in the establishment of the sterility barrier. In this context, genetic incompatibilities involving the duplicated F-box genes as putative candidates, and a possible strengthening step involving a chromosomal inversion that increases the genetic linkage between the factors involved in the epistatic interaction are suspected to participate in the reproductive barrier between Asian and African rice species. The knowledge generated by these comparative approaches contributes to a better understanding of the general evolution of postzygotic reproductive barriers in plants. Additionally, it allows considering new breeding strategies aiming unlocking the genetic potential of *O. glaberrima* for the improvement of the Asian rice. Additional efforts still remain necessary to confirm the candidate genes and to identify the molecular mechanism that controls the *S_1_* postzygotic barrier.

## Materials and Methods

### Sequence analysis and gene annotation method

*O. glaberrima* cv. CG14 BAC sequencing was done by the Sanger method. Sequence analysis was done as previously described [27]. Briefly, coding regions were predicted *ab initio* using the FGENESH program [50] and then confirmed by comparative analysis with annotated genes models and proteins in *O. sativa* cv. Nipponbare, downloaded from the TIGR database [51]. Predicted gene structures were manually evaluated by alignment with rice EST and full-length cDNA (FLcDNA) public sequences [52]. Detailed analysis was performed with the EMBOSS Analysis software [53] and the physical map diagram was drawn using gff2ps software [54]. Putative transposable elements (TEs) were first identified and annotated by RepeatMasker searches (http://www.repeatmasker.org) against local databases of rice TEs downloaded from the REPBASE [55], from the TIGR repeat database [56], and RetrOryza [57], and finally manually corrected. *De novo* prediction of TEs was performed according to structure of the different classes of TEs. The final annotation of the BAC sequences was performed using the Artemis tool [58], and the comparison with the Nipponbare genome was accomplished using dot-plot alignments of the Dotter software [59]. Nucleotide and amino-acid alignments were carried out using ClustalX [60].

### Molecular marker analysis

Genetic markers were designed from the comparison of the Nipponbare sequence with its orthologous CG14 sequence as previously described [27], and evaluated in four *O. sativa*×*O. glaberrima* BC_1_F_1_ populations developed from our previous work [27]. PCR reactions were carried as described [61], with an annealing temperature and magnesium concentration optimized for each primer pair (Table S6). Separation of the PCR products was carried in 4% agarose and revealed with Ethidium Bromide for polymorphisms greater than 12 bp, and in a Li-Cor sequencer (Li-Cor Biosciences) for smaller polymorphisms, using a M13 tail tag (IRD700 and IRD800).

### Detection of a chromosomal inversion in *Oryza* species by mapping BAC end sequence pairs

Public BESs from 9 *Oryza* species (*O. sativa*, *O. rufipogon*, *O. glaberrima*, *O. nivara*, *O. punctata*, *O. minuta*, *O. officinalis*, *O. alta* and *O. australiensis*) developed in the frame of the Oryza Map Alignment Project (OMAP, http://www.omap.org) were downloaded from AGI web site (http://www.genome.arizona.edu/stc/rice/) [33]. BACs were mapped onto the *O. glaberrima S_1_* region by aligning BES pairs using BLASTN. BACs overlapping the chromosomal inversion breakpoints (as indicated by the alignment of the two BES of each BAC, inside and outside the inverted region, within a distance <300,000 bp) were filtered, and the orientation of both BESs relative to the *O. glaberrima S_1_* genomic region was analyzed.

### Orthologous sequence comparisons

The orthologous CG14 *S_1_* regions were identified by BLASTN against the *O. sativa* ssp. *japonica* cv Nipponbare pseudomolecules (release v. 6.1) downloaded from the MSU Rice Genome Annotation Project web site [51], and against the *O. sativa* ssp. *indica* 93-11 downloaded from the Beijing Genomic Institute web site (http://rice.genomics.org.cn/rice2/link/download.jsp). Sequence comparisons were carried out using the Dotter program [59], the Artemis Comparison Tool [62], and the EMBOSS package. The downloaded *O. sativa* sequences were re-annotated for genes and TEs with similar approaches used to annotate the *O. glaberrima* segment. To study microcollinearity between orthologous *O. glaberrima* and *O. sativa* sequences, the nucleotide sequences of non-TE coding genes were extracted for each segment and used as queries for BLAST alignments between each other to generate a distance matrix. Microcollinearity relationships were displayed using GenomePixelizer software (http://www.atgc.org/GenomePixelizer/).

### Calculation of nonsynonymous and synonymous nucleotide substitution rates

Orthologous *O. sativa* and *O. glaberrima* annotated coding regions were aligned using the Needle tools [53] to estimate the degree of gene structure conservations. Orthologous genes with clear distinct annotated gene structure were removed from further analysis. Calculations for nonsynonymous and synonymous nucleotides substitution rate were done as previously described [27]. Identical analyses were carried out with two control loci recently sequenced in *O. glaberrima*: *ADH* [18] from chromosome 11 (positions 5.598–5.750 Mbp) and *MOC1* from chromosome 6 (positions 24.25–24,40 Mbp) [35]. A non-parametric statistical test (Kruskal–Wallis analysis of variance) was used to analyze the homogeneity of Ka and Ks data between the *S_1_* region and the *ADH* and *MOC1* loci. *P*<0.05 was considered to be statistically significant to report non homogenous data.

## Supporting Information

Figure S1 **Schematic representation of microcollinearity relationships between** ***O. glaberrima*** **cv. GC14 and** ***O. sativa*** **cv. Nipponbare** ***S_1_*** **regions.** Colored boxes indicate positions and orientation of non-TE genes along axes representing the CG14 (upper segment) and Nipponbare (lower segment) *S_1_* regions. Colored lines linking boxes symbolize high identity relationships between one, or several genes from CG14 and Nipponbare. Red boxes indicate genes lacking orthologs. Blue and green boxes represent the positions of duplicated genes in Nipponbare compared to CG14. Orange boxes indicate the positions of genes contained in the inversion. The positions of *S_1_A*, *S_1_* and *S_1_B* loci are indicated along the horizontal axis of CG14. Identified gene families in CG14 as classified in Table 1 are indicated below the diagram.
(TIF)

Figure S2 **Mapping of pairs of BAC end sequences (BES) from seven** ***Oryza*** **species spanning the** ***O. glaberrima*** **inversion breakpoints.** Pairs of BAC end sequences (BES) were mapped on the *O. glaberrima S_1_* regions. BACs spanning the inversion breakpoints were symbolized on the *O. glaberrima* physical map as horizontal lines limited by colored arrows, representing orientations of BES (blue arrows for sense orientations and red arrows for antisense orientations). The name of each mapped BAC is indicated below each horizontal line. BACs limited by two BES in opposite orientation indicate similar organization of the inversion compared to *O. glaberrima*, while BACs limited by two BES in identical orientation suggest a different orientation compared to *O. glaberrima*. A tree in the left of the figure symbolizes the evolutionary relationships of the *Oryza* species used in this analysis as described in Ge *et al.*, 1999 (Proc Natl Acad Sci U S A, 96:14400–14405).
(EPS)

Figure S3 **Comparison between structural variations identified between orthologous** ***S_1_*** **regions in CG14 and Nipponbare, and the genetic map of the** ***S_1_*** **locus between** ***O. sativa*** **and** ***O. glaberrima*** **.** Structural variations between orthologous *S_1_* regions (left) compared with the genetic map obtained from the *O. sativa*×*O. glaberrima* 779 BC_1_F_1_ plants previously described [27]. Marker positions in the CG14 physical map are shown. Blue frames on the physical maps of *O. glaberrima* cv. CG 14 and *O. sativa* ssp. *japonica* cv. Nipponbare indicate the position of the chromosomal inversion.
(EPS)

Figure S4 **Frequencies of Ka and Ks values between orthologous coding sequences in the** ***O. glaberrima*** **and** ***O. sativa S_1_*** **,** ***ADH*** **and** ***MOC1*** **regions.** Distribution of the Ka and Ks values of the *S_1_* (**A**), *ADH* (**B**) and *MOC1* (**C**) orthologous regions from *O. sativa* ssp. *japonica* cv. Nipponbare and *O. glaberrima* cv. CG14.
(EPS)

Figure S5 **Amino-acid alignment between duplicated F-box proteins in the** ***S_1_*** **orthologous regions.** Alignment of predicted amino-acid sequences of F-box genes in the *S_1_* and *S_1_A* loci from *O. sativa* ssp. *japonica* cv. Nipponbare (OSj), *O. sativa* ss. *indica* cv. 93-11 (OSi) et *O. glaberrima* cv. CG14 (OG). Blue box indicates the identified F-box domain. The conserved Leucine amino acids in the Leucine rich repeat regions are underlined in grey. *S_1_A*-OSj^p^ designates the duplicated and partial F-box protein in the Nipponbare *S_1_A* locus.
(EPS)

Figure S6 **Phylogenetic relationships among the** ***S_1_*** **and** ***S_1_A*** **orthologous F-box proteins.** Phylogenetic tree of the *S_1_* and *S_1_A* orthologous F-box genes from *O. sativa* ssp. *japonica* cv. Nipponbare (OSj), *O. sativa* ssp. *indica* cv. 93-11 (OSi) and *O. glaberrima* cv. CG14 (OG). The unrooted tree was generated by the neighbor-joining method using ClustalX program. Numbers indicates bootstrap values with 1000 replicates.
(EPS)

Table S1 **List of identified genes in the 813 kb of the** ***O. glaberrima*** **cv. CG14** ***S_1_*** **regions.**
(DOC)

Table S2 **List of the different types of TE found in the** ***O. glaberrima S_1_*** **regions.**
(DOC)

Table S3 **List of identified pack-MULEs and enclosed genes in the** ***O. glaberrima*** **cv. CG14** ***S_1_*** **regions.**
(DOC)

Table S4 **Sequence comparison between orthologous coding sequences in the** ***O. glaberrima*** **cv. CG14 and** ***O. sativa*** **cv. Nipponbare** ***ADH*** **and** ***MOC1*** **regions.**
(DOC)

Table S5 **Sequence comparison between orthologous coding sequences in the** ***O. glaberrima*** **cv. CG14 and** ***O. sativa*** **cv. Nipponbare** ***S_1_*** **regions.**
(DOC)

Table S6 **New molecular markers designed in the** ***S_1_*** **regions.**
(DOC)
