# Supplementary material for: Patterns of Sequence Divergence and Evolution of the S1 Orthologous Regions between Asian and African Cultivated Rice Species
Source: PLoS One. 2011 Mar 10;6(3):e17726. doi: 10.1371/journal.pone.0017726 (PMC3053390; doi:10.1371/journal.pone.0017726)
Supplement: Table S1 — List of identified genes in the 813 kb of the O. glaberrima cv. CG14 S1 regions. (DOC) [file pone.0017726.s007.doc]

Table S1 - List of identified genes in the 813 kb of the *O. glaberrima* cv. CG14 *S_1_* regions

| **Locus** | **Gene Name** | **Putative Nipponbare orthologous gene** | **Putative Function** | **Protein domain** | **Best BLASTX homology (Swiss prot)** | **Best BLASTN homology** |
| --- | --- | --- | --- | --- | --- | --- |
| - | OG-BBa0093E08.1^p^ | LOC_Os06g04450 | Putative Sec1-like protein | pfam00995, Sec1, Sec1 family | Q5VNU3 *O. sativa* Probable protein transport Sec1b (0.0) | AK100230 *O. sativa* flcDNA (0.0) |
| - | OG-BBa0093E08.2 | LOC_Os06g04460 | Putative protein | cd03407, Band_7_4, A subgroup of the band 7 domain of flotillin (reggie) like proteins. | P16148 *Lupinus polyphyllus* Protein PPLZ12  (2e-69) | AK102678 *O. sativa* flcDNA (0.0) |
| - | OG-BBa0093E08.3* | no orthologous gene | Putative protein | / | / | AK120604 *O. sativa* flcDNA  (5e-41) |
| - | OG-BBa0093E08.5 | no orthologous gene | Putative protein | pfam03478, Protein of unknown function (DUF295) | / | AP004234 *O. sativa* chr. 6 (0.0) |
| - | OG-BBa0093E08.6 | LOC_Os06g04470 | Putative protein | / | / | AK073180 *O. sativa* flcDNA (0.0) |
| - | OG-BBa0093E08.7 | LOC_Os06g04480 | Putative protein | / | Q56XJ7 *A. thaliana* protein At4g22760 (2e-09) | AK109132 *O. sativa* flcDNA  (3e-167) |
| - | OG-BBa0093E08.8 | LOC_Os06g04490 | Putative protein | / | / | AK288873 *O. sativa* flcDNA  (1e-166) |
| - | OG-BBa0093E08.9 | LOC_Os06g04500 | Putative cornichon protein | pfam03311, Cornichon protein | P53173 *S. cerevisiae* ER-derived vesicles protein ERV14 (5e-20) | CT829434 *O. sativa* flcDNA (0.0) |
| - | OG-BBa0093E08.10 | LOC_Os06g04510 | Putative Enolase | cd03313, Enolase; PRK00077, phosphopyruvate hydratase | P26301 *Z. mais* Enolase 1 | AK069316 *O. sativa* flcDNA (0.0) |
| - | OG-BBa0093E08.11 | LOC_Os06g04520 | Putative protein | / | Q3UMB9 *M. musculus* UPF0681 protein KIAA1033 (2e-37) | AK065081 *O. sativa* flcDNA (0.0) |
| - | OG-BBa0093E08.12 | LOC_Os06g04530 | Putative protease | pfam00574, CLP_protease | Q9XJ36 *Arabidopsis* ATP-dependent Clp protease proteolytic subunit (6e-90) | AK104375 *O. sativa* flcDNA (0.0) |
| - | OG-BBa0093E08.13 | LOC_Os06g04540 | Putative protein | cl00720, Domain of unknown function (DUF296) | Q9S7C9 *Arabidopsis* Putative DNA-binding protein ESCAROLA (4e-44) | AK107405 *O. sativa* flcDNA (0.0) |
| - | OG-BBa0093E08.14 | LOC_Os06g04560 | Putative Armadillo protein | cd00106, Kinesin motor domain; cd00020, Armadillo/beta-catenin-like repeats | Q5VQ09 *O. sativa* Armadillo repeat-containing kinesin-like protein (0.0) | AK242714 *O. sativa* flcDNA (0.0) |
| - | OG-BBa0093E08.145*^PM^ | LOC_Os06g04530 | Hypothetical protein | / | / | AP002071 *O. sativa* chr. 6 (5e-172) |
| - | OG-BBa0093E08.15 | LOC_Os06g04580 | Putative protein | cl02094, YEATS family | Q755P0 *Eremothecium gossypii* Protein AF-9 homolog (6e-31) | AK109053 *O. sativa* flcDNA (0.0) |
| *S_1_A* | OG-BBa0093E08.16 | LOC_Os06g04590 | Putative Auxin responsive protein | pfam02519, Auxin responsive protein | P33083 *G. max* Auxine induced protein 6b (1e-7) | AP003708 *O. sativa* chr. 6 (0.0) |
| *S_1_A* | OG-BBa0093E08.17 | LOC_Os06g04600 | Putative protein | cl00935, Brix, Brix domain | Q5R631 *Pongo abelii* Ribosome production factor 1 (2e-73) | AK072896 *O. sativa* flcDNA (0.0) |
| *S_1_A* | OG-BBa0093E08.18 | LOC_Os06g04610 | Putative Ribosome Binding factor | cl00542, RBFA, Ribosome-binding factor A | O65693 *A. thaliana* Probable ribosome-binding factor A (4e-56) | AK099752 *O. sativa* flcDNA (0.0) |
| *S_1_A* | OG-BBa0093E08.19 | LOC_Os06g04620 | Putative Epimerase | pfam01370, NAD dependent epimerase/dehydratase family | P93031 *A. thaliana* GDP-mannose 4,6 dehydratase 2 (4e-157) | AK073547 *O. sativa* flcDNA (0.0) |
| *S_1_A* | OG-BBa0093E08.195^PM^ | LOC_Os06g04630 | Putative protein | cl00321, AAT_I, Aspartate aminotransferase (AAT) superfamily | O07051, *Aeromonas jandei* L-allo-threonine aldolase (e-7) | AP003708 *O. sativa* chr. 6 (1e-135) |
| *S_1_A* | OG-BBa0093E08.20 | LOC_Os06g04640 | Putative transcription factor | cl02556, Bromodomain | Q9FT54 *A. thaliana* Transcription factor GTE6 | AK250146 *H. vulgare* cDNA (0.0) |
| *S_1_A* | OG-BBa0093E08.21 | LOC_Os06g04650 | Putative methionine sulfoxide reductase | cl00366, Peptide methionine sulfoxide reductase | P08761 *D. melanogaster* Peptide methionine sulfoxide reductase (7e-30) | CT832709 *O. sativa* flcDNA (0.0) |
| *S_1_A* | OG-BBa0093E08.22 | LOC_Os06g04660 | Putative protein | cl01206, 2OG-Fe(II) oxygenase superfamily | Q6P6C2 *H. sapiens* Alkylated DNA repair protein alkb homolog 5 (5e-12) | AK073705 *O. sativa* flcDNA (0.0) |
| *S_1_A* | OG-BBa66E18.1 | LOC_Os06g04670 | Putative CAF1 ribonuclease | cl09229, CAF1 family ribonuclease | O74856 *S. pombe* Poly(A) ribonuclease pop2 (4e-27) | AP003708 *O. sativa* chr. 6 (0.0) |
| *S_1_A* | OG-BBa66E18.2 | LOC_Os06g04680 | Putative protein | pfam03141, DUF248, Putative methyltransferase | / | AK069536 *O. sativa* flcDNA (0.0) |
| *S_1_A* | OG-BBa66E18.3 | LOC_Os06g04699 | Putative protein | / | / | AK105368 *O. sativa* flcDNA (0.0) |
| *S_1_A* | OG-BBa66E18.4 | LOC_Os06g04690 | Putative F-box protein | / | Q9SMT9 *A. thaliana* FBD-associated F-box protein  (5e-4) | AK073027 O. sativ flcDNA (0.0) |
| *S_1_A* | OG-BBa66E18.42*^PM^ | LOC_Os06g04760 | Hypothetical protein | / | / | AP003708 *O. sativa* chr. 6 (0.0) |
| *S_1_A* | OG-BBa66E18.45^PM^ | no orthologous gene | Hypothetical protein | / | / | AP003708 *O. sativa* chr. 6 (0.0) |
| *S_1_A* | OG-BBa66E18.5 | LOC_Os06g04780 | Putative protein | pfam09747, Coiled-coil domain containing protein (DUF2052) | Q96F63 *H. sapiens* Coiled-coil domain-containing protein 9 (4e-16) | AK106220 *O. sativa* flcDNA (0.0) |

Table S1 - Continuation

| **Locus** | **Gene Name** | **Putative Nipponbare orthologous gene** | **Putative Function** | **Protein domain** | **Best BLASTX homology (Swiss prot)** | **Best BLASTN homology** |
| --- | --- | --- | --- | --- | --- | --- |
| *S_1_A* | OG-BBa66E18.6 | LOC_Os06g04790 | Puttive HAD phosphatase | cl00375, Haloacid Dehalogenase-like Hydrolases | P27061 *S. lycopersicum* Acid phosphatase (6e-87) | AK058604 *O. sativa* flcDNA (0.0) |
| *S_1_A* | OG-BBa66E18.7 | LOC_Os06g04800 | Putative proteasome subunit | cd03762, proteasome beta type-6 subunit. | Q8LD27 *A. thaliana* Proteasome subunit beta type-6  (5e-97) | AK104152 *O. sativa* flcDNA (0.0) |
| *S_1_A* | OG-BBa66E18.8 | LOC_Os06g04810 | Putative LRR protein | pfam08263, Leucine rich repeat N-terminal domain | Q9LYN8 *A. thaliana* Leucine-rich repeat receptor protein kinase EXS (2e-56) | AP003708 *O. sativa* chr. 6 (0.0) |
| *S_1_A* | OG-BBa17A24.1 | LOC_Os06g04820 | Putative methyltransferase | cl01306, Leucine carboxyl methyltransferase | Q6P4Z6 *Rattus norvegicus* Leucine carboxyl methyltransferase 1 (7e-43) | AK063371 *O. sativa* flcDNA (0.0) |
| *S_1_A* | OG-BBa17A24.2 | LOC_Os06g04830 | Putative LRR protein | COG4886, Leucine-rich repeat (LRR) protein | Q9FL28 *A. thaliana* LRR receptor-like serine/threonine-protein kinase (4e-72) | AK287701 *O. sativa* flcDNA (0.0) |
| *S_1_A* | OG-BBa17A24.25*^PM^ | no orthologous gene | Hypothetical protein | / | / | AP004329 *O. sativa* chr. 6 (0.0) |
| *S_1_A* | OG-BBa17A24.3 | LOC_Os06g04840 | Putative LRR protein | pfam08263, Leucine rich repeat N-terminal domain | Q9FL28 *A. thaliana* LRR receptor-like serine/threonine-protein kinase (2e-69) | AK100368 *O. sativa* flcDNA (0.0) |
| *S_1_A* | OG-BBa17A24.4 | LOC_Os06g04850 | Putative protein | cd00086, homeodomain | Q5VPE5 *O. sativa* Homeobox-leucine zipper protein HOX28 (3e-75) | AK059116 *O. sativa* flcDNA (0.0) |
| *S_1_A* | OG-BBa17A24.5 | LOC_Os06g04860 | Hypothetical protein | / | / | AP003708 *O. sativa* chr. 6 (2e-45) |
| *S_1_A* | OG-BBa17A24.6 | LOC_Os06g04870 | Putative protein | cd00086, homeodomain, | Q5VPE3 *O. sativa* Homeobox-leucine zipper protein HOX2 (5e-96) | AK105150 *O. sativa* flcDNA (0.0) |
| *S_1_A* | OG-BBa17A24.7 | LOC_Os06g04880 | Putative serine/Threonine protein kinase | cd00180, Serine/Threonine protein kinases, catalytic domain; cd01989, The N-terminal domain of Eukaryotic Serine Threonine kinases | Q9LU47 *A. thaliana* Putative U-box domain-containing protein 53 (3e-164) | AK065683 *O. sativa* flcDNA (0.0) |
| *S_1_A* | OG-BBa17A24.8 | LOC_Os06g04889 | Putative protein | / | / | AK058823 *O. sativa* flcDNA  (1e-99) |
| *S_1_A* | OG-BBa17A24.9 | LOC_Os06g04900 | Putative protein | cl09119, Major Facilitator Superfamily (MFS) | Q07423 Ricinus communis Hexose carrier protein HEX6 (9e-147) | NM_001063291 *O. sativa* Os06g0141000 (0.0) |
| *S_1_A* | OG-BBa17A24.10 | LOC_Os06g04910 | Putative NADH pyrophosphatase protein | pfam09296, NUDIX-like; cd03429, NADH_pyrophosphatase | Q94A82 *A. thaliana* NADH pyrophosphatase NUDT19 (8e-69) | NM_001063292 *O. sativa* Os06g0141100 (0.0) |
| *S_1_A* | OG-BBa0049I08.2 | LOC_Os06g04920 | Putative Zinc finger protein | pfam00641, Zn-finger | O13801 *S. pombe* RNA-binding protein (2e-9) | CT830197 *O. sativa* flcDNA (0.0) |
| *S_1_A* | OG-BBa0049I08.3 | LOC_Os06g04930 | Putative ENOD93 protein | pfam03386, Early nodulin 93 ENOD93 protein | Q02921 *G. max* Early nodulin 93 (2e-11) | AK121184 *O. sativa* flcDNA  (2e-131) |
| *S_1_A* | OG-BBa0049I08.4^PM^ | no orthologous gene | Hypothetical protein | cd06163 zinc metalloproteases | P73714 *Synechocystis* (1e-9) | AC136219 *O. sativa* BAC (0.0) |
| *S_1_A* | OG-BBa0049I08.45*^PM^ | no orthologous gene | Hypothetical protein | / | / | AC109601 *O. sativa* BAC (8e-41) |
| *S_1_* | OG-BBa0049I08.5 | LOC_Os06g04940 | Putative ENOD93 protein | pfam03386, Early nodulin 93 ENOD93 protein | Q02921 *Glycine max* Early nodulin 93 (8e-20) | AK122162 *O. sativa* flcDNA  (2e-148) |
| *S_1_* | OG-BBa0049I08.6 | LOC_Os06g04950 | Putative ENOD93 protein | pfam03386, Early nodulin 93 ENOD93 protein | Q02921 *G. max* Early nodulin 93 (4e-14) | AK121791 *O. sativa* flcDNA  (1e-157) |
| *S_1_* | OG-BBa0049I08.7 | no orthologous gene | Hypothetical protein | / | / | / |
| *S_1_* | OG-BBa0049I08.75^PM^ | no orthologous gene | Hypothetical protein | / | / | Ak107277 *O. sativa* flcDNA  (1e-115) |
| *S_1_* | OG-BBa0049I08.8 | LOC_Os06g04970 | Putative protein | / | Q9SH88 *A. thaliana* Ribosome biogenesis regulatory protein (3e-48) | NM_001063299 *O. sativa* Os06g0142000 (0.0) |
| *S_1_* | OG-BBa0049I08.85* | no orthologous gene | Hypothetical protein | / | / | AP002838 *O. sativa* BAC (2e-156) |
| *S_1_* | OG-BBa0049I08.9*^PM^ | no orthologous gene | Hypothetical protein | / | / | AP004090 *O. sativa* BAC (8e-69) |
| *S_1_* | OG-BBa0049I08.10*^PM^ | no orthologous gene | Hypothetical protein | / | P47927 *A. thaliana* Floral homeotic protein APETALA 2 (4e-8) | AC121363 *O. sativa* BAC (3e-128) |
| *S_1_* | OG-BBa0049I08.11 | LOC_Os06g04980 | Putative F-box protein | / | Q9FJT2 *A. thaliana* F-box/FBD/LRR-repeat (5e-4) | AK073027 *O. sativa* fcDNA (0.0) |

Table S1 - Continuation

| **Locus** | **Gene Name** | **Putative Nipponbare orthologous gene** | **Putative Function** | **Protein domain** | **Best BLASTX homology (Swiss prot)** | **Best BLASTN homology** |
| --- | --- | --- | --- | --- | --- | --- |
| *S_1_* | OG-BBa0049I08.12 | LOC_Os06g04990 | Putative ENOD93 protein | pfam03386, Early nodulin 93 ENOD93 protein | Q02921 *G. max* Early nodulin 93 (1e-17) | AK122162 *O. sativa* flcDNA  (2e-175) |
| *S_1_B* | OG-BBa0049I08.13 | LOC_Os06g05000 | Putative ENOD93 protein | pfam03386, Early nodulin 93 ENOD93 protein | Q02921 *G. max* Early nodulin 93 (1e-14) | CT837557 *O. sativa* flcDNA  (4e-177) |
| *S_1_B* | OG-BBa0049I08.14 | LOC_Os06g05010 | Putative ENOD93 protein | pfam03386, Early nodulin 93 ENOD93 protein | Q02921 *G. max* Early nodulin 93 (1e-17) | CT827995 *O. sativa* flcDNA  (1e-158) |
| *S_1_B* | OG-BBa0049I08.15 | LOC_Os06g05020 | Putative ENOD93 protein | pfam03386, Early nodulin 93 ENOD93 protein | Q02921 *G. max* Early nodulin 93 (1e-17) | CT827995 *O. sativa* flcDNA  (9e-173) |
| *S_1_B* | OG-BBa0049I08.16 | LOC_Os06g05050^C^ | Putative Serine/Threonine protein kinases | cd00180, Serine/Threonine protein kinases | Q9LMN7 *A. thaliana* Wall-associated receptor kinase 5 (5e-130) | NM_001063304 *O. sativa* Os06g0142500 (0.0) |
| *S_1_B* | OG-BBa0049I08.17 | LOC_Os06g05060 | Putative Early flowering protein | / | O82804 *A. thaliana* Protein EARLY FLOWERING 3 (1e-55) | NM_001063305 *O. sativa* Os06g0142600 (0.0) |
| *S_1_B* | OG-BBa0049I08.18 | LOC_Os06g05070 | Putative Serine/Threonine protein kinases | cd00180, Serine/Threonine protein kinases | O23081 *A. thaliana* Cysteine-rich receptor-like protein kinase 41 (2e-57) | AP000399 *O. sativa* BAC (0.0) |
| *S_1_B* | OG-BBa0049I08.19 | LOC_Os06g05080 | Putative cytochrome oxidase | cd00924, Cytochrome c oxidase subunit Vb | P00428 *Bos taurus* Cytochrome c oxidase subunit 5B, mitochondrial (6e-14) | CT834467 *O. sativa* flcDNA (0.0) |
| *S_1_B* | OG-BBa0049I08.20 | LOC_Os06g05090 | Putative methyltransferase protein | cd02440, S-adenosylmethionine-dependent methyltransferases | Q9SNQ2 *O. sativa* protein arginine N-methyltransferase (0.0) | AK103971 *O. sativa* flcDNA (0.0) |
| *S_1_B* | OG-BBa0049I08.21 | LOC_Os06g05100 | Putative 1-deoxy-D-xylulose-5-phosphate synthase | cd02007, Thiamine pyrophosphate (TPP) family, DXS subfamily, TPP-binding module | Q38854 *A. thaliana* 1-deoxy-D-xylulose-5-phosphate synthase, chloroplastic (0.0) | AK121920 *O. sativa* flcDNA (0.0) |
| *S_1_B* | OG-BBa0049I08.22 | LOC_Os06g05110 | Putative Superoxide dismutase | PRK10543, superoxide dismutase; pfam02777, Iron/manganese superoxide dismutases | P22302 *Nicotiana plumbaginifolia* Superoxide dismutase [Fe], chloroplastic (5e-60) | AK071301 *O. sativa* flcDNA (0.0) |
| *S_1_B* | OG-BBa0049I08.23 | LOC_Os06g05120 | Putative protein | / | / | AK061597 *O. sativa* flcDNA  (9e-80) |
| *S_1_B* | OG-BBa0049I08.24 | LOC_Os06g05130 | Putative Myristoyl-acyl carrier protein thioesterase | cd00586, 4-hydroxybenzoyl-CoA thioesterase (4HBT) | Q9SQI3 *Gossypium hirsutum* Myristoyl-acyl carrier protein thioesterase, chloroplastic (6e-146) | AK120946 *O. sativa* flcDNA (0.0) |
| *S_1_B* | OG-BBa0049I08.25 | LOC_Os06g05140 | Putative PPR protein | pfam01535, PPR repeat | O49287 *A. thaliana* Putative pentatricopeptide repeat-containing protein (2e-93) | AP003487 *O. sativa* BAC (0.0) |
| *S_1_B* | OG-BBa0049I08.26 | LOC_Os06g05150^C^ | Putative protein | pfam03371, PRP38 family | Q80SY5 *M. musculus* Pre-mRNA-splicing factor 38B (2e-40) | AP003487 *O. sativa* BAC (0.0) |
| *S_1_B* | OG-BBa0049I08.27 | LOC_Os06g05160 | Putative sulfate transporter | pfam00916,Sulfate transporter family | Q9LW86 *A. thaliana* Probable sulfate transporter (1e-141) | AK067270 *O. sativa* flcDNA (0.0) |
| *S_1_B* | OG-BBa0045G15.1 | LOC_Os06g05180 | Putative Coatomer protein | pfam04053, Coatomer WD associated region; cd00200, WD40 | Q5VQ78 *O. sativa* Coatomer subunit beta'-1 | AK111584 *O. sativa* flcDNA (0.0) |
| *S_1_B* | OG-BBa0045G15.2 | LOC_Os06g05190 | Putative BRCA1 protein | cl00038, Breast Cancer Suppressor Protein (BRCA1), carboxy-terminal domain. | Q60596 *M. musculus* DNA repair protein XRCC1 (2e-23) | AB292807 *O. sativa* xrcc1 mRNA (0.0) |
| *S_1_B* | OG-BBa0088O22.1 | LOC_Os06g05200 | Putative Zinc finger protein | cd00162, RING-finger | / | AP003487 *O. sativa* chr. 6 (0.0) |
| *S_1_B* | OG-BBa0088O22.2 | LOC_Os06g05209 | Putative pectate lyase protein | cl01593, Pectate lyase | P40973 *Lilium longiflorum* Pectate lyase (1e-126) | AK100191 *O. sativa* flcDNA (0.0) |
| *S_1_B* | OG-BBa0088O22.3 | LOC_Os06g05260 | Putative pectate lyase protein | cl01593, Pectate lyase | P40973 *Lilium longiflorum* Pectate lyase (1e-142) | AK070509 *O. sativa* flcDNA (0.0) |
| *S_1_B* | OG-BBa0088O22.4 | LOC_Os06g05250 | Putative GTP-binding protein | PRK05433, GTP-binding protein LepA; cd03699, lepA_II; cd03709, lepA_C | Q8N442 *H. sapiens* GTP-binding protein GUF1 (0.0) | AK103630 *O. sativa* flcDNA (0.0) |
| *S_1_B* | OG-BBa0088O22.5 | LOC_Os06g05240 | Putative carboxypeptidase | cl10475, Zinc carboxypeptidase | Q9JHW1 *Ratus norvegicus* Carboxypeptidase D (5e-69) | BT038203 *Z. mais* flcDNA (0.0) |
| *S_1_B* | OG-BBa0088O22.6 | LOC_Os06g05220 | Hypothetical protein | pfam03080, *Arabidopsis* proteins of unknown function | / | AP003487 *O. sativa* chr. 6 (6e-96) |
| *S_1_B* | OG-BBa0088O22.7 | LOC_Os06g05272 | Putative pectate lyase protein | cl01593, Pectate lyase | P40973 *Lilium longiflorum* Pectate lyase (2e-134) | AK100191 *O. sativa* flcDNA (0.0) |

Table S1 - Continuation

| **Locus** | **Gene Name** | **Putative Nipponbare orthologous gene** | **Putative Function** | **Protein domain** | **Best BLASTX homology (Swiss prot)** | **Best BLASTN homology** |
| --- | --- | --- | --- | --- | --- | --- |
| *S_1_B* | OG-BBa0088O22.8 | LOC_Os06g05284 | Putative Transferase | pfam02458, Transferase family | Q9ZWR8 *Gentiana triflora* Anthocyanin 5-aromatic acyltransferase (2e-16) | AP003487 *O. sativa* chr. 6 (0.0) |
| *S_1_B* | OG-BBa0088O22.9 | LOC_Os06g05300 | Putative Transferase | pfam02458, Transferase | Q9ZWR8 *Gentiana triflora* Anthocyanin 5-aromatic acyltransferase (4e-14) | AP003487 *O. sativa* chr. 6 (0.0) |
| *S_1_B* | OG-BBa0088O22.10 | LOC_Os06g05310 | Putative Transferase | pfam02458, Transferase | Q9ZWR8 *Gentiana triflora* Anthocyanin 5-aromatic acyltransferase (2e-16) | AP003487 *O. sativa* chr. 6 (0.0) |
| *S_1_B* | OG-BBa0088O22.11 | LOC_Os06g05320 | Putative Transferase | pfam02458, Transferase | / | AK106249 *O. sativa* flcDNA (0.0) |
| *S_1_B* | OG-BBa0088O22.12 | LOC_Os06g05340 | Putative AP2 proyein | cd00018, AP2 | Q6X5Y6 *A. thaliana* Ethylene-responsive transcription factor WRI1 (3e-74) | AP003487 *O. sativa* chr. 6 (0.0) |
| *S_1_B* | OG-BBa0088O22.13 | LOC_Os06g05350 | Putative Whirly transcription factor protein | pfam08536, Whirly transcription factor | / | CT836194 *O. sativa* flcDNA (0.0) |
| *S_1_B* | OG-BBa0088O22.14 | LOC_Os06g05359 | Putative protein | cl09099, P-loop containing Nucleoside Triphosphate Hydrolases | Q9STE7 *A. thaliana* Putative disease resistance RPP13-like protein (5e-75) | AK120452 *O. sativa* flcDNA (0.0) |
| *S_1_B* | OG-BBa0088O22.15 | LOC_Os06g05368 | Putative protein | / | / | NM_001063327 *O. sativa* Os06g0146200 (1e-151) |
| *S_1_B* | OG-BBa0088O22.16 | LOC_Os06g05380 | Putative WRKY protein | pfam03106, WRKY DNA -binding domain | Q9LXG8 *A. thaliana* Probable WRKY transcription factor 72 (4e-53) | BK005076 *O. sativa* WRKY transcription factor (0.0) |
| *S_1_B* | OG-BBa0088O22.17 | LOC_Os06g05390 | Putative protein | pfam09353, Domain of unknown function (DUF1995) | / | AK101052 *O. sativa* flcDNA (0.0) |
| *S_1_B* | OG-BBa0088O22.18 | LOC_Os06g05400 | Putative Iron-sulphur protein | cl00400, Iron-sulphur cluster biosynthesis | Q9XIK3 *A. thaliana* Iron-sulfur assembly protein IscA (5e-47) | CT832410 *O. sativa* flcDNA (0.0) |
| *S_1_B* | OG-BBa0088O22.19 | LOC_Os06g05410 | Hypothetical protein | / | / | AP003767 *O. sativa* chr. 6 (0.0) |
| *S_1_B* | OG-BBa0056F23.1 | LOC_Os06g05420 | Putative protein | / | / | AP003767 *O. sativa*, chr. 6 (3e-173) |
| *S_1_B* | OG-BBa0056F23.2 | LOC_Os06g05424 | Putative protein | / | / | AK071352 *O. sativa* flcDNA (4e-138) |
| *S_1_B* | OG-BBa0056F23.3 | LOC_Os06g05430 | Putative protein | / | / | AP003767 *O. sativa*, chr. 6 (3e-169) |
| *S_1_B* | OG-BBa0056F23.4 | LOC_Os06g05440 | Putative protein | / | / | AP003767 *O. sativa*, chr. 6 (3e-178) |
| *S_1_B* | OG-BBa0056F23.5 | LOC_Os06g05470 | Putative protein | / | / | AP003767 *O. sativa*, chr. 6 (0.0) |
| *S_1_B* | OG-BBa0056F23.6 | LOC_Os06g05480 | Putative protein | / | / | AP003767 *O. sativa*, chr. 6 (0.0) |
| *S_1_B* | OG-BBa0056F23.7 | LOC_Os06g05510 | Putative protein | / | / | AP003767 *O. sativa*, chr. 6 (0.0) |
| *S_1_B* | OG-BBa0056F23.8 | LOC_Os06g05520 | Putative protein kinase | cd00180, Serine/Threonine protein kinases, catalytic domain | Q9S7U9 *A. thaliana* Mitogen-activated protein kinase kinase 2 (7e-124) | AK111598 *O. sativa* flcDNA (0.0) |
| *S_1_B* | OG-BBa0056F23.9 | LOC_Os06g05530 | Putative protein | / | / | CT828147 *O. sativa* flcDNA (0.0) |
| *S_1_B* | OG-BBa0056F23.10 | LOC_Os06g05540 | Hypothetical protein | / | / | AP003767 *O. sativa*, chr. 6 (0.0) |
| *S_1_B* | OG-BBa0056F23.11 | LOC_Os06g05550 | GDSL esterase/lipase protein | cd01837, SGNH_plant_lipase_like | Q0WPI9 *A. thaliana* GDSL esterase/lipase (2e-95) | CT831308 *O. sativa* flcDNA (0.0) |
| *S_1_B* | OG-BBa0056F23.12 | LOC_Os06g05560 | Putative protein | pfam03478, Protein of unknown function (DUF295) | / | AP008212 *O. sativa*, chr. 6 (0.0) |
| *S_1_B* | OG-BBa0056F23.13 | LOC_Os06g05580 | Putative F-box protein | pfam03478, Protein of unknown function (DUF295) | O49315 *A. thaliana* Putative F-box protein (2e-6) | AP008212 *O. sativa*, chr. 6 (0.0) |
| *S_1_B* | OG-BBa0056F23.14* | LOC_Os06g05590 | Putative F-box protein | / | / | AP008212 *O. sativa*, chr. 6 (0.0) |
| *S_1_B* | OG-BBa0056F23.15 | LOC_Os06g05600 | Putative F-box protein | pfam03478, Protein of unknown function (DUF295); cl02535, F-box, F-box domain | / | AK287605 *O. sativa* flcDNA (0.0) |
| *S_1_B* | OG-BBa0056F23.16 | LOC_Os06g05610 | Putative F-box protein | pfam03478, Protein of unknown function (DUF295); cl02535, F-box, F-box domain | / | AK107542 *O. sativa* flcDNA (0.0) |
| *S_1_B* | OG-BBa0056F23.17* | LOC_Os06g05620 | Putative F-box protein | / | / | AP008212 *O. sativa*, chr. 6 (0.0) |
| *S_1_B* | OG-BBa0056F23.18 | LOC_Os06g05630 | GDSL esterase/lipase protein | cd01837, SGNH_plant_lipase_like | Q0WPI9 *A. thaliana* GDSL esterase/lipase (2e-95) | AK099064 *O. sativa* flcDNA (0.0) |
| *S_1_B* | OG-BBa0056F23.19 | LOC_Os06g05640 | Putative protein | / | / | AP008212 *O. sativa*, chr. 6 (0.0) |

Table S1 - Continuation

| **Locus** | **Gene Name** | **Putative Nipponbare orthologous gene** | **Putative Function** | **Protein domain** | **Best BLASTX homology (Swiss prot)** | **Best BLASTN homology** |
| --- | --- | --- | --- | --- | --- | --- |
| *S_1_B* | OG-BBa0056F23.20 | LOC_Os06g05650 | Putative protein | / | / | AP008212 *O. sativa*, chr. 6 (3e-93) |
| *S_1_B* | OG-BBa0041E07.1 | LOC_Os06g05660 | Putative Nucleosome protein | pfam00956, Nucleosome assembly protein (NAP) | Q4U0Y4 *Xenopus laevis* Nucleosome assembly protein 1-like (6e-38) | AK071177 *O. sativa* flcDNA (0.0) |
| *S_1_B* | OG-BBa0041E07.2 | LOC_Os06g05670 | Putative protein | / | / | AK102827 *O. sativa* flcDNA  (3e-179) |
| *S_1_B* | OG-BBa0041E07.3 | LOC_Os06g05690 | putative Cystein synthase protein | COG0031, CysK, Cysteine synthase | P32260 *Spinacia oleracea* Cysteine synthase (1e-91) | AK243242 *O. sativa* flcDNA (0.0) |
| *S_1_B* | OG-BBa0041E07.4 | LOC_Os06g05700 | putative Cystein synthase protein | COG0031, CysK, Cysteine synthase | Q9XEA8 *O. sativa* Cysteine synthase (9e-89) | AK072457 *O. sativa* flcDNA (0.0) |
| *S_1_B* | OG-BBa0041E07.5 | LOC_Os06g05710 | Putative protein | / | / | AP008212 *O. sativa*, chr. 6 (0.0) |
| *S_1_B* | OG-BBa0041E07.6 | LOC_Os06g05720 | Putative protein | / | / | AK068890 *O. sativa* flcDNA (0.0) |
| *S_1_B* | OG-BBa0041E07.7 | LOC_Os06g05730 | Putative protein | / | / | AK072773 *O. sativa* flcDNA (0.0) |
| *S_1_B* | OG-BBa0041E07.8 | LOC_Os06g05740 | Putative protein | / | / | AK287795 *O. sativa* flcDNA (0.0) |
| *S_1_B* | OG-BBa0041E07.9 | LOC_Os06g05750 | Putative transferase protein | pfam02458, Transferase family | O24645 *Dianthus caryophyllus* benzoyltransferase protein (2e-12) | AK063346 *O. sativa* flcDNA (0.0) |
| *S_1_B* | OG-BBa0041E07.10 | LOC_Os06g05760 | Putative ubiquitin protein | cd01769, UBL | O14399 *S. pombe* Ubiquitin-like protein (e-5) | AP008212 *O. sativa*, chr. 6 (0.0) |
| *S_1_B* | OG-BBa0041E07.11* | LOC_Os06g05770 | Hypothetical protein | / | / | / |
| *S_1_B* | OG-BBa0041E07.12 | LOC_Os06g05790 | Putative transferase protein | pfam02458, Transferase family | / | AP008212 *O. sativa*, chr. 6 (0.0) |
| *S_1_B* | OG-BBa0041E07.13 | LOC_Os06g05800 | Putative protein | cd00590, RRM (RNA recognition motif) | Q9WVB0 *M. musculus* RNA-binding protein with multiple splicing (6e-12) | AK068636 *O. sativa* flcDNA (0.0) |
| *S_1_B* | OG-BBa0041E07.14 | LOC_Os06g05804 | Putative trafficking protein | cl02131, Sedlin_N | Q54UU1 *Dictyostelium discoideum* Trafficking protein particle complex (3e-31) | AK241792 *O. sativa* flcDNA (0.0) |
| *S_1_B* | OG-BBa0041E07.15 | LOC_Os06g05820 | Putative protein | cl09099, P-loop NTPase; PRK11823, DNA repair protein RadA | P37572 *Bacillus subtilis* DNA repair protein radA homolog (2e-92) | AB111516 *O. sativa* OsRadA mRNA (0.0) |
| *S_1_B* | OG-BBa0041E07.16 | LOC_Os06g05830 | Putative protein kinase | cd00180, S_TKc, Serine/Threonine protein kinases; pfam00069, Pkinase | O24585 *Z.maize* Putative receptor protein kinase CRINKLY4 (4e-48) | AK106925 *O. sativa* flcDNA (0.0) |
| *S_1_B* | OG-BBa0041E07.17 | LOC_Os06g05860 | Putative phosphofructokinase protein | cl00204, PFK, Phosphofructokinase | Q94AA4 *A. thaliana* 6-phosphofructokinase 3 (0.0) | BT063863 *Z. mais* flcDNA mRNA (0.0) |
| *S_1_B* | OG-BBa0041E07.18 | LOC_Os06g05870 | Putative protein phosphatase | cl00053, PTPc Protein tyrosine phosphatases | P0C089 *Ratus norvegicus* Protein-tyrosine phosphatase mitochondrial 1 (1e-25) | BT068138 *Z. mais* flcDNA clone (4e-176) |
| *S_1_B* | OG-BBa0041E07.19 | LOC_Os06g05880 | Putative profilin protein | cd00148, Profilin binds actin monomers | Q5VMJ3 *O. sativa* Profilin LP04 (6e-75) | CT833929 *O. sativa* flcDNA (0.0) |
| *S_1_B* | OG-BBa0041E07.20 | LOC_Os06g05890 | Putative zinc-finger protein | cd00021, BBOX, B-Box-type zinc finger; zinc binding domain (CHC3H2) | Q9SYM2 *A. thaliana* Putative salt tolerance-like protein (1e-43) | AK104083 *O. sativa* flcDNA (0.0) |
| *S_1_B* | OG-BBa0041E07.21 | LOC_Os06g05900 | Putative Methylase protein | COG2227, 2-polyprenyl-3-methyl-5-hydroxy-6-metoxy-1,4-benzoquinol methylase | O49354 *A. thaliana* Hexaprenyldihydroxybenzoate methyltransferase (4e-109) | AK064315 *O. sativa* flcDNA (0.0) |
| *S_1_B* | OG-BBa0041E07.22 | LOC_Os06g05910 | Putative methyltransferases | cd02440, S-adenosylmethionine-dependent methyltransferases | O49354 *A. thaliana* Hexaprenyldihydroxybenzoate methyltransferase (4e-49) | AK064640 *O. sativa* flcDNA (0.0) |
| *S_1_B* | OG-BBa0041E07.23 | LOC_Os06g05920 | Putative PRR protein | pfam01535, PPR repeat | Q9FIX3 *A. thaliana* Pentatricopeptide repeat-containing protein (4e-170) | AP008212 *O. sativa*, chr. 6 (0.0) |
| *S_1_B* | OG-BBa0041E07.24 | LOC_Os06g05930 | Putative protein | / | / | CT828676 *O. sativa* flcDNA (0.0) |
| *S_1_B* | OG-BBa0041E07.25 | LOC_Os06g05940 | Putative protein | cl02724, DUF821 | A0NDG6 *Anopheles gambiae* CAP10 family protein AGAP004267 (2e-18) | AK109934 *O. sativa* flcDNA (0.0) |
| *S_1_B* | OG-BBa0041E07.26 | LOC_Os06g05950 | Putative protein | / | / | AK099578 *O. sativa* flcDNA (0.0) |
| *S_1_B* | OG-BBa0041E07.27 | LOC_Os06g05960 | Putative protein | / | / | AP008212 *O. sativa*, chr. 6 (3e-153) |
| *S_1_B* | OG-BBa0041E07.28 | LOC_Os06g05980 | Putative protein | cl01037, Integral membrane protein DUF6 | Q10354 *S. pombe* Uncharacterized transporter (8e-48) | AK072625 *O. sativa* flcDNA (0.0) |
| *S_1_B* | OG-BBa0041E07.29^p^ | LOC_Os06g05990 | / | / | / | / |

p: partial gene; *: Pseudogene; PM: Pack-MULE.
