# Supplementary material for: Patterns of Sequence Divergence and Evolution of the S1 Orthologous Regions between Asian and African Cultivated Rice Species
Source: PLoS One. 2011 Mar 10;6(3):e17726. doi: 10.1371/journal.pone.0017726 (PMC3053390; doi:10.1371/journal.pone.0017726)
Supplement: Table S2 — List of the different types of TE found in the O. glaberrima S1 regions. (DOC) [file pone.0017726.s008.doc]

Table S2 - List of the different types of TE found in the *O. glaberrima* *S_1_* regions

| **Class** | **Sub class** | **Types** | **Numbers** |
| --- | --- | --- | --- |
| I | Non LTR retrotransposons | LINEs | 5 |
|  |  | SINEs | 13 |
|  | LTR retrotransposons | Solo LTR | 11 |
|  |  | Complete | 4 |
|  |  | partial | 4 |
| II | MITEs | Tourist | 5 |
|  |  | Stowaway | 65 |
|  |  | Other | 128 |
|  | Transposons | - | 138 |
