# Supplementary material for: Patterns of Sequence Divergence and Evolution of the S1 Orthologous Regions between Asian and African Cultivated Rice Species
Source: PLoS One. 2011 Mar 10;6(3):e17726. doi: 10.1371/journal.pone.0017726 (PMC3053390; doi:10.1371/journal.pone.0017726)
Supplement: Table S3 — List of identified pack-MULEs and enclosed genes in the O. glaberrima cv. CG14 S1 regions. (DOC) [file pone.0017726.s009.doc]

Table S3 - List of identified pack-MULEs and enclosed genes in the *O. glaberrima* cv. CG14 *S_1_* regions

| **Name of transposon** | **Coordinates (bp)** | **Enclosed gene** | **Putative Function** | **Protein domain** | **Best BLASTX homology (swiss prot)** | **Best BLASTN homology** |
| --- | --- | --- | --- | --- | --- | --- |
| Pack-MULE#1 | 96761-98626 | **OG-BBa0093E08.145** | Hypothetical protein; Pseudogene | / | / | AP002071 *O. sativa* chr. 6 (5e-172) |
| Pack-MULE#2 | 130291-132132 | **OG-BBa0093E08.195** | Putative protein | cl00321, AAT_I, Aspartate aminotransferase (AAT) superfamily | O07051, Aeromonas jandei L-allo-threonine aldolase (e-7) | AP003708 *O. sativa* chr. 6 (1e-135) |
| Pack-MULE#3 | 165290-167410 | **OG-BBa0066E18.42** | Hypothetical protein; Pseudogene | / | / | AP003708 *O. sativa* chr. 6 (0.0) |
| Pack-MULE#4 | 172505-173829 | **OG-BBa0066E18.45** | Hypothetical protein | / | / | AP003708 *O. sativa* chr. 6 (0.0) |
| Pack-MULE#5 | 201668-203440 | **OG-BBa17A24.25** | Hypothetical protein; Pseudogene | / | / | AP004329 *O. sativa* chr. 6 (0.0) |
| Pack-MULE#6 | 270384-271956 | **OG-BBa0049I08.4** | Hypothetical protein | cd06163 zinc metalloproteases | P73714 Synechocystis (1e-9) | AC136219 *O. sativa* BAC (0.0) |
| Pack-MULE#7 | 272132-273102 | **OG-BBa0049I08.45** | Hypothetical protein; Pseudogene | / | / | AC109601 *O. sativa* BAC (8e-41) |
| Pack-MULE#8 | 294743-296002 | **OG-BBa0049I08.75** | Hypothetical protein | / | / | Ak107277 *O. sativa* flcDNA (1e-115) |
| Pack-MULE#9 | 307632-308718 | **OG-BBa0049I08.9** | Hypothetical protein; Pseudogene | / | / | AP004090 *O. sativa* BAC (8e-69) |
| Pack-MULE#10 | 310525-311981 | **OG-BBa0049I08.10** | Hypothetical protein; Pseudogene | / | P47927 *A. thaliana* Floral homeotic protein APETALA 2 (4e-8) | AC121363 *O. sativa* BAC (3e-128) |
