# Supplementary material for: Patterns of Sequence Divergence and Evolution of the S1 Orthologous Regions between Asian and African Cultivated Rice Species
Source: PLoS One. 2011 Mar 10;6(3):e17726. doi: 10.1371/journal.pone.0017726 (PMC3053390; doi:10.1371/journal.pone.0017726)
Supplement: Table S4 — Sequence comparison between orthologous coding sequences in the O. glaberrima cv. CG14 and O. sativa cv. Nipponbare ADH and MOC1 regions. (DOC) [file pone.0017726.s010.doc]

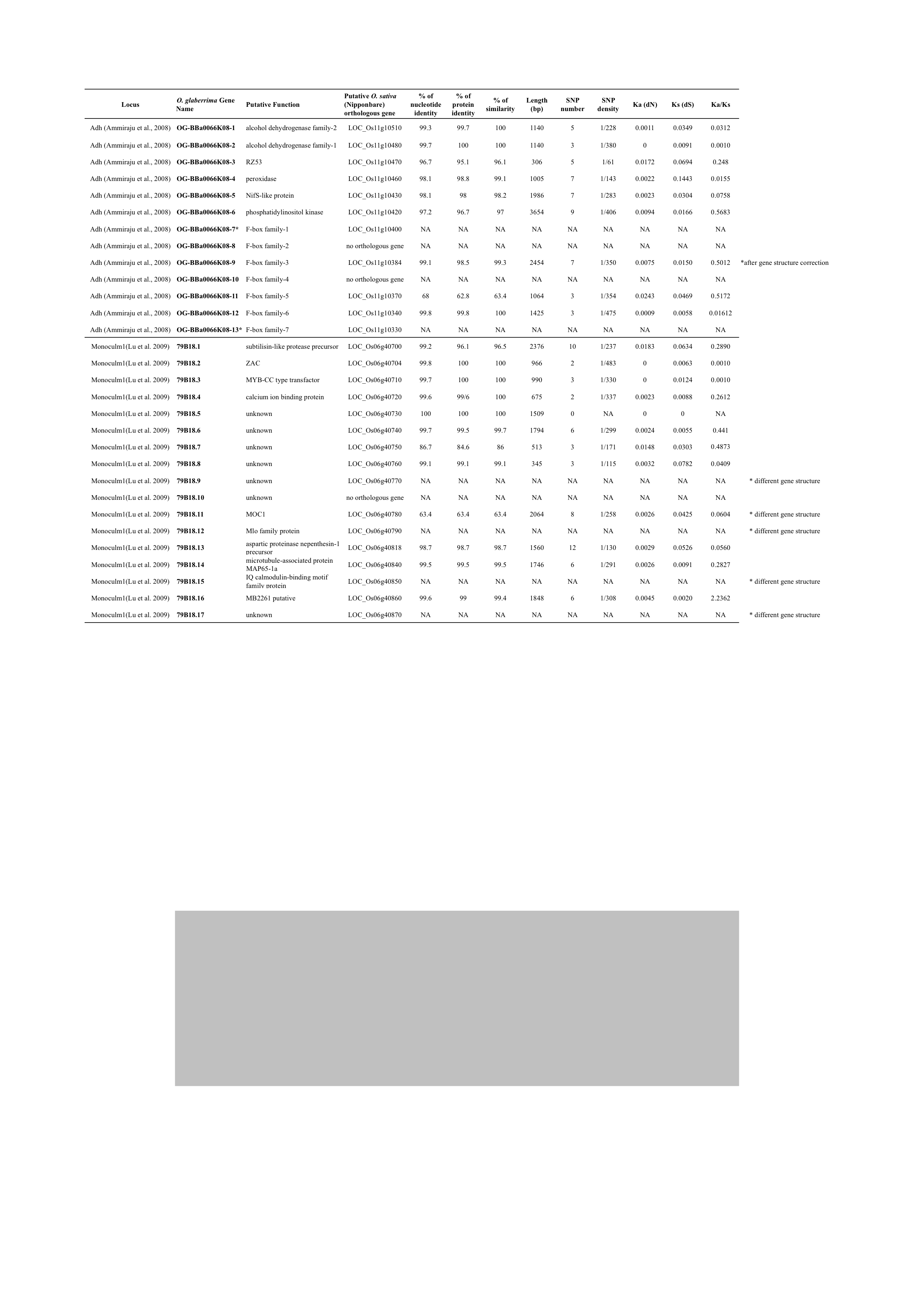
**Table S4 - Sequence comparison between orthologous coding sequences in the *O. glaberrima* cv. CG14 and *O. sativa* cv. Nipponbare *ADH* and *MOC1* regions**
