# Supplementary material for: Patterns of Sequence Divergence and Evolution of the S1 Orthologous Regions between Asian and African Cultivated Rice Species
Source: PLoS One. 2011 Mar 10;6(3):e17726. doi: 10.1371/journal.pone.0017726 (PMC3053390; doi:10.1371/journal.pone.0017726)
Supplement: Table S5 — Sequence comparison between orthologous coding sequences in the O. glaberrima cv. CG14 and O. sativa cv. Nipponbare S1 regions. (DOC) [file pone.0017726.s011.doc]

**Table S5 - Sequence comparison between orthologous coding sequences in the *O. glaberrima* cv. CG14 and *O. sativa* cv. Nipponbare *S_1_*** regions

| **Locus** | ***O. glaberrima* Gene Name** | **Putative Function** | **Putative *O. sativa* (Nipponbare) orthologous gene** | **% of nucleotide identity** | **% of protein identity** | **% of similarity** | **Length (bp)** | **Ka (dN)** | **Ks (dS)** | **Ka/Ks** |
| --- | --- | --- | --- | --- | --- | --- | --- | --- | --- | --- |
| - | **OG-BBa0093E08.1^p^** | Putative Sec1-like protein | LOC_Os06g04450 | NA | NA | NA | NA | NA | NA | NA |
| - | **OG-BBa0093E08.2** | Putative protein | LOC_Os06g04460 | 100 | 100 | 100 | 867 | 0.0000 | 0.0000 | 0.3504 |
| - | **OG-BBa0093E08.3*** | Putative protein | no orthologous gene | NA | NA | NA | NA | NA | NA | NA |
| - | **OG-BBa0093E08.5** | Putative protein | no orthologous gene | NA | NA | NA | NA | NA | NA | NA |
| - | **OG-BBa0093E08.6** | Putative protein | LOC_Os06g04470 | 94.4 | 92.2 | 95.1 | 1233 | 0.0372 | 0.0914 | 0.4065 |
| - | **OG-BBa0093E08.7** | Putative protein | LOC_Os06g04480 | 95 | 95.2 | 96.8 | 378 | 0.0104 | 0.1226 | 0.0845 |
| - | **OG-BBa0093E08.8** | Putative protein | LOC_Os06g04490 | 87.1 | 85.5 | 86.2 | 455 | 0.0167 | 0.1534 | 0.1092 |
| - | **OG-BBa0093E08.9** | Putative cornichon protein | LOC_Os06g04500 | 99.5 | 100 | 100 | 408 | 0.0000 | 0.0190 | 0.0010 |
| - | **OG-BBa0093E08.10** | Putative Enolase | LOC_Os06g04510 | 99.2 | 99.3 | 99.3 | 1341 | 0.0030 | 0.0252 | 0.120 |
| - | **OG-BBa0093E08.11** | Putative protein | LOC_Os06g04520 | 81.9 | 81.6 | 81.6 | 1995 | 0.003 | 0.0033 | 0.9003 |
| - | **OG-BBa0093E08.12** | Putative protease | LOC_Os06g04530 | 99.7 | 99.3 | 99.3 | 894 | 0.0030 | 0.0048 | 0.6253 |
| - | **OG-BBa0093E08.13** | Putative protein | LOC_Os06g04540 | 99.1 | 98.8 | 98.8 | 990 | 0.0037 | 0.0189 | 0.195 |
| - | **OG-BBa0093E08.14** | Putative Armadillo protein | LOC_Os06g04560 | 99.9 | 100 | 100 | 2676 | 0.0000 | 0.0024 | 0.001 |
| - | **OG-BBa0093E08.145*^PM^** | Hypothetical protein | LOC_Os06g04530 | NA | NA | NA | NA | NA | NA | NA |
| - | **OG-BBa0093E08.15** | Putative protein | LOC_Os06g04580 | 100 | 100 | 100 | 951 | 0.0000 | 0.0000 | 0.4546 |
| ***S_1_A*** | **OG-BBa0093E08.16** | Putative Auxin responsive protein | LOC_Os06g04590 | 100 | 100 | 100 | 360 | 0.0000 | 0.0000 | 0.001 |
| ***S_1_A*** | **OG-BBa0093E08.17** | Putative protein | LOC_Os06g04600 | 96.7 | 83.5 | 85.2 | 1216 | 0.0739 | 0.1474 | 0.5011 |
| ***S_1_A*** | **OG-BBa0093E08.18** | Putative Ribosome Binding factor | LOC_Os06g04610 | 99.8 | 100 | 100 | 630 | 0.0000 | 0.0059 | 0.0010 |
| ***S_1_A*** | **OG-BBa0093E08.19** | Putative Epimerase | LOC_Os06g04620 | 99.7 | 99.7 | 100 | 1128 | 0.001 | 0.023 | 0.0420 |
| ***S_1_A*** | **OG-BBa0093E08.195^PM^** | Putative protein | LOC_Os06g04630 | 100 | 100 | 100 | 498 | 0.0000 | 0.0000 | 0.2965 |
| ***S_1_A*** | **OG-BBa0093E08.20** | Putative transcription factor | LOC_Os06g04640 | 99.8 | 100 | 100 | 1083 | 0.0000 | 0.0053 | 0.0010 |
| ***S_1_A*** | **OG-BBa0093E08.21** | Putative methionine sulfoxide reductase | LOC_Os06g04650 | 99.7 | 100 | 100 | 765 | 0.0000 | 0.0102 | 0.0010 |
| ***S_1_A*** | **OG-BBa0093E08.22** | Putative protein | LOC_Os06g04660 | 99.5 | 99 | 99.2 | 1851 | 0.0046 | 0.0059 | 0.777 |
| ***S_1_A*** | **OG-BBa66E18.1** | Putative CAF1 ribonuclease | LOC_Os06g04670 | 99 | 98.8 | 99.1 | 1008 | 0.0022 | 0.0261 | 0.0845 |
| ***S_1_A*** | **OG-BBa66E18.2** | Putative protein | LOC_Os06g04680 | 99.2 | 99.2 | 99.5 | 1152 | 0.001 | 0.013 | 0.0783 |
| ***S_1_A*** | **OG-BBa66E18.3** | Putative protein | LOC_Os06g04699 | 96.9 | 94 | 96 | 1053 | 0.0276 | 0.0571 | 0.4831 |
| ***S_1_A*** | **OG-BBa66E18.4** | Putative F-box protein | LOC_Os06g04690 | 96.9 | 94 | 96.9 | 1560 | 0.0299 | 0.0369 | 0.8120 |
| ***S_1_A*** | **OG-BBa66E18.42*^PM^** | Hypothetical protein | LOC_Os06g04760 | NA | NA | NA | NA | NA | NA | NA |
| ***S_1_A*** | **OG-BBa66E18.45** | Hypothetical protein | no orthologous gene | NA | NA | NA | NA | NA | NA | NA |
| ***S_1_A*** | **OG-BBa66E18.5** | Putative protein | LOC_Os06g04780 | 98.2 | 98.1 | 98.4 | 936 | 0.0054 | 0.0487 | 0.1109 |
| *S_1_A* | **OG-BBa66E18.6** | Puttive HAD phosphatase | LOC_Os06g04790 | 96.3 | 94.9 | 98 | 882 | 0.0266 | 0.1089 | 0.244 |
| *S_1_A* | **OG-BBa66E18.7** | Putative proteasome subunit | LOC_Os06g04800 | 98.5 | 98.8 | 99.6 | 741 | 0.0056 | 0.0427 | 0.1318 |
| *S_1_A* | **OG-BBa66E18.8** | Putative LRR protein | LOC_Os06g04810 | 86.9 | 79.8 | 86.3 | 2892 | 0.1114 | 0.2014 | 0.5528 |
| *S_1_A* | **OG-BBa17A24.1** | Putative methyltransferase | LOC_Os06g04820 | 97.5 | 97.1 | 98 | 1026 | 0.0119 | 0.054 | 0.2194 |
| *S_1_A* | **OG-BBa17A24.2** | Putative LRR protein | LOC_Os06g04830 | 88 | 82.7 | 87.2 | 2955 | 0.0696 | 0.0839 | 0.8295 |
| *S_1_A* | **OG-BBa17A24.25*^PM^** | Hypothetical protein | no orthologous gene | NA | NA | NA | NA | NA | NA | NA |
| *S_1_A* | **OG-BBa17A24.3** | Putative LRR protein | LOC_Os06g04840 | 95 | 92.4 | 93.2 | 2754 | 0.0229 | 0.0351 | 0.6528 |
| *S_1_A* | **OG-BBa17A24.4** | Putative protein | LOC_Os06g04850 | 98.3 | 97.3 | 98.1 | 780 | 0.0063 | 0.0001 | NA |
| *S_1_A* | **OG-BBa17A24.5** | Hypothetical protein | LOC_Os06g04860 | 99.5 | 100 | 100 | 195 | 0.0000 | 0.0156 | 0.001 |
| *S_1_A* | **OG-BBa17A24.6** | Putative protein | LOC_Os06g04870 | 99.5 | 99 | 99.4 | 930 | 0.0025 | 0.0000 | NA |
| *S_1_A* | **OG-BBa17A24.7** | Putative serine/Threonine protein kinase | LOC_Os06g04880 | 99.9 | 99.9 | 99.9 | 2421 | 0.0006 | 0.0032 | 0.1766 |
| *S_1_A* | **OG-BBa17A24.8** | Putative protein | LOC_Os06g04889 | 100 | 100 | 100 | 207 | 0.0000 | 0.0000 | 0.0761 |
| *S_1_A* | **OG-BBa17A24.9** | Putative protein | LOC_Os06g04900 | 99.9 | 99.8 | 99.8 | 1563 | 0.0007 | 0.0072 | 0.0983 |
| *S_1_A* | **OG-BBa17A24.10** | Putative NADH pyrophosphatase protein | LOC_Os06g04910 | 99.3 | 99 | 99.3 | 1224 | 0.0025 | 0.0000 | NA |
| *S_1_A* | **OG-BBa0049I08.2** | Putative Zinc finger protein | LOC_Os06g04920 | 99.3 | 99.3 | 100 | 438 | 0.0028 | 0.0266 | 0.1063 |
| *S_1_A* | **OG-BBa0049I08.3** | Putative ENOD93 protein | LOC_Os06g04930 | 61.3 | 60.9 | 62.2 | NA | NA | NA | NA |
| *S_1_A* | **OG-BBa0049I08.4** | Hypothetical protein | no orthologous gene | NA | NA | NA | NA | NA | NA | NA |
| *S_1_A* | **OG-BBa0049I08.45*^PM^** | Hypothetical protein | no orthologous gene | NA | NA | NA | NA | NA | NA | NA |
| *S_1_* | **OG-BBa0049I08.5** | Putative ENOD93 protein | LOC_Os06g04940 | 65 | 54.1 | 59.5 | NA | NA | NA | NA |
| *S_1_* | **OG-BBa0049I08.6** | Putative ENOD93 protein | LOC_Os06g04950 | 89.1 | 76.8 | 78.4 | 376 | NA | NA | NA |
| *S_1_* | **OG-BBa0049I08.7** | Hypothetical protein | no orthologous gene | NA | NA | NA | NA | NA | NA | NA |
| *S_1_* | **OG-BBa0049I08.75^PM^** | Hypothetical protein | no orthologous gene | NA | NA | NA | NA | NA | NA | NA |
| *S_1_* | **OG-BBa0049I08.8** | Putative protein | LOC_Os06g04970 | 98.6 | 97.7 | 98.4 | 2073 | 0.0112 | 0.0237 | 0.4728 |

Table S5 - Continuation

| **Locus** | ***O. glaberrima* Gene Name** | **Putative Function** | **Putative *O. sativa* (Nipponbare) orthologous gene** | **% of nucleotide identity** | **% of protein identity** | **% of similarity** | **Length (bp)** | **Ka (dN)** | **Ks (dS)** | **Ka/Ks** |
| --- | --- | --- | --- | --- | --- | --- | --- | --- | --- | --- |
| *S_1_* | **OG-BBa0049I08.85*** | Hypothetical protein | no orthologous gene | NA | NA | NA | NA | NA | NA | NA |
| *S_1_* | **OG-BBa0049I08.9* ^PM^** | Hypothetical protein | no orthologous gene | NA | NA | NA | NA | NA | NA | NA |
| *S_1_* | **OG-BBa0049I08.10*^PM^** | Hypothetical protein | no orthologous gene | NA | NA | NA | NA | NA | NA | NA |
| *S_1_* | **OG-BBa0049I08.11** | Putative F-box protein | LOC_Os06g04980 | 98.1 | 96.5 | 97.7 | 1542 | 0.0173 | 0.0198 | 0.8715 |
| *S_1_* | **OG-BBa0049I08.12** | Putative ENOD93 protein | LOC_Os06g04990 | 99.7 | 100 | 100 | 348 | 0.0000 | 0.0142 | 0.001 |
| *S_1_B* | **OG-BBa0049I08.13** | Putative ENOD93 protein | LOC_Os06g05000 | 100 | 100 | 100 | 348 | 0.0000 | 0.0000 | NA |
| *S_1_B* | **OG-BBa0049I08.14** | Putative ENOD93 protein | LOC_Os06g05010 | 99.4 | 100 | 100 | 351 | 0.000025 | 0.0260 | 0.001 |
| *S_1_B* | **OG-BBa0049I08.15** | Putative ENOD93 protein | LOC_Os06g05020 | 99.1 | 98.3 | 99.1 | 348 | 0.0074 | 0.0143 | 0.5175 |
| *S_1_B* | **OG-BBa0049I08.16** | Putative Serine/Threonine protein kinases | LOC_Os06g05050^C^ | 92 | 87.8 | 92.1 | 2291 | 0.0447 | 0.0571 | 0.7828 |
| *S_1_B* | **OG-BBa0049I08.17** | Putative Early flowering protein | LOC_Os06g05060 | 99.2 | 98.8 | 99.1 | 2283 | 0.0057 | 0.0155 | 0.3669 |
| *S_1_B* | **OG-BBa0049I08.18** | Putative Serine/Threonine protein kinases | LOC_Os06g05070 | 97.6 | 97.6 | 98.5 | 1386 | 0.0081 | 0.1700 | 0.0465 |
| *S_1_B* | **OG-BBa0049I08.19** | Putative cytochrome oxidase | LOC_Os06g05080 | 99.8 | 99.4 | 99.4 | 465 | 0.0032 | 0.0000 | NA |
| *S_1_B* | **OG-BBa0049I08.20** | Putative methyltransferase protein | LOC_Os06g05090 | 98.8 | 98.4 | 99 | 1143 | 0.0071 | 0.0299 | 0.2366 |
| *S_1_B* | **OG-BBa0049I08.21** | Putative 1-deoxy-D-xylulose-5-phosphate synthase | LOC_Os06g05100 | 99.9 | 99.6 | 99.7 | 2169 | 0.0018 | 0.0000 | NA |
| *S_1_B* | **OG-BBa0049I08.22** | Putative Superoxide dismutase | LOC_Os06g05110 | 99.9 | 99.6 | 100 | 768 | 0.0016 | 0.0000 | NA |
| *S_1_B* | **OG-BBa0049I08.23** | Putative protein | LOC_Os06g05120 | NA | NA | NA | NA | NA | NA | NA |
| *S_1_B* | **OG-BBa0049I08.24** | Putative Myristoyl-acyl carrier protein thioesterase | LOC_Os06g05130 | 97.4 | 99 | 99 | 1284 | 0.0044 | 0.0300 | 0.1457 |
| *S_1_B* | **OG-BBa0049I08.25** | Putative PPR protein | LOC_Os06g05140 | 99.8 | 99.6 | 99.6 | 2292 | 0.0018 | 0.0037 | 0.4789 |
| *S_1_B* | **OG-BBa0049I08.26** | Putative protein | LOC_Os06g05150^C^ | 98 | 97.2 | 97.5 | 1296 | 0.0070 | 0.0048 | 1.471 |
| *S_1_B* | **OG-BBa0049I08.27** | Putative sulfate transporter | LOC_Os06g05160^p^ | 99.2 | 99.4 | 99.6 | 2013 | 0.0018 | 0.0373 | 0.0489 |
| *S_1_B* | **OG-BBa0045G15.1** | Putative Coatomer protein | LOC_Os06g05180 | 99.3 | 98.7 | 98.8 | 2736 | 0.0054 | 0.0098 | 0.5528 |
| *S_1_B* | **OG-BBa0045G15.2** | Putative BRCA1 protein | LOC_Os06g05190 | 99.6 | 99.1 | 99.7 | 1044 | 0.0042 | 0.0032 | 1.3209 |
| *S_1_B* | **OG-BBa0088O22.1** | Putative Zinc finger protein | LOC_Os06g05200 | 91.4 | 89.8 | 90.3 | 618 | 0.0086 | 0.0414 | 0.2088 |
| *S_1_B* | **OG-BBa0088O22.2** | Putative pectate lyase protein | LOC_Os06g05209 | 76.3 | 76.7 | 76.9 | 1362 | 0.0020 | 0.2075 | 0.0098 |
| *S_1_B* | **OG-BBa0088O22.3** | Putative pectate lyase protein | LOC_Os06g05260 | 99 | 98.4 | 98.4 | 1353 | 0.0041 | 0.0209 | 0.1942 |
| *S_1_B* | **OG-BBa0088O22.4** | Putative GTP-binding protein | LOC_Os06g05250 | 99.8 | 99.7 | 99.8 | 1992 | 0.0014 | 0.0035 | 0.4173 |
| *S_1_B* | **OG-BBa0088O22.5** | Putative carboxypeptidase | LOC_Os06g05240 | 99.4 | 98.3 | 98.3 | 1053 | 0.0076 | 0.0001 | NA |
| *S_1_B* | **OG-BBa0088O22.6** | Hypothetical protein | LOC_Os06g05220 | 72.1 | 67.1 | 68.8 | 728 | 0.0526 | 0.0927 | 0.5674 |
| *S_1_B* | **OG-BBa0088O22.7** | Putative pectate lyase protein | LOC_Os06g05272 | 76.2 | 76.7 | 76.9 | 1362 | 0.0020 | 0.2409 | 0.0084 |
| *S_1_B* | **OG-BBa0088O22.8** | Putative Transferase | LOC_Os06g05284 | 99 | 98.4 | 98.8 | 1473 | 0.0057 | 0.0198 | 0.2873 |
| *S_1_B* | **OG-BBa0088O22.9** | Putative Transferase | LOC_Os06g05300 | 99.3 | 99.2 | 99.2 | 1479 | 0.0008 | 0.0053 | 0.1513 |
| *S_1_B* | **OG-BBa0088O22.10** | Putative Transferase | LOC_Os06g05310 | 96.9 | 95.5 | 97.1 | 1353 | 0.0185 | 0.0380 | 0.4872 |
| *S_1_B* | **OG-BBa0088O22.11** | Putative Transferase | LOC_Os06g05320 | 65.6 | 53.8 | 55.2 | 1458 | 0.0763 | 0.2235 | 0.3414 |
| *S_1_B* | **OG-BBa0088O22.12** | Putative AP2 proyein | LOC_Os06g05340 | 99.3 | 99.5 | 99.5 | 1200 | 0.0000 | 0.0083 | 0.0010 |
| *S_1_B* | **OG-BBa0088O22.13** | Putative Whirly transcription factor protein | LOC_Os06g05350 | 99.4 | 98.9 | 99.3 | 819 | 0.0052 | 0.0086 | 0.6043 |
| *S_1_B* | **OG-BBa0088O22.14** | Putative protein | LOC_Os06g05359 | 99.2 | 98.1 | 98.5 | 3081 | 0.0093 | 0.0065 | 1.4143 |
| *S_1_B* | **OG-BBa0088O22.15** | Putative protein | LOC_Os06g05368 | 72.7 | 55 | 59.7 | 432 | NA | NA | NA |
| *S_1_B* | **OG-BBa0088O22.16** | Putative WRKY protein | LOC_Os06g05380 | 99.6 | 99.4 | 99.4 | 1584 | 0.0025 | 0.0089 | 0.2758 |
| *S_1_B* | **OG-BBa0088O22.17** | Putative protein | LOC_Os06g05390 | 99.8 | 99.7 | 99.7 | 1023 | 0.0013 | 0.0042 | 0.3090 |
| *S_1_B* | **OG-BBa0088O22.18** | Putative Iron-sulphur protein | LOC_Os06g05400 | 99.6 | 99.5 | 100 | 561 | 0.0025 | 0.0066 | 0.3816 |
| *S_1_B* | **OG-BBa0088O22.19** | Hypothetical protein | LOC_Os06g05410 | 87.5 | 86.2 | 87.7 | 393 | 0.0693 | 0.1361 | 0.5093 |
| *S_1_B* | **OG-BBa0056F23.1** | Putative protein | LOC_Os06g05420 | 99.4 | 98.3 | 98.3 | 348 | 0.0083 | 0.0001 | NA |
| *S_1_B* | **OG-BBa0056F23.2** | Putative protein | LOC_Os06g05424 | 100 | 100 | 100 | 276 | 0.0000 | 0.0000 | 0.0010 |
| *S_1_B* | **OG-BBa0056F23.3** | Putative protein | LOC_Os06g05430 | 95.5 | 94.1 | 95.8 | 357 | 0.0262 | 0.1303 | 0.2012 |
| *S_1_B* | **OG-BBa0056F23.4** | Putative protein | LOC_Os06g05440 | 99.2 | 98.3 | 99.2 | 360 | 0.0063 | 0.0355 | 0.1768 |
| *S_1_B* | **OG-BBa0056F23.5** | Putative protein | LOC_Os06g05470 | 100 | 100 | 100 | 459 | 0.0000 | 0.0000 | 0.0010 |
| *S_1_B* | **OG-BBa0056F23.6** | Putative protein | LOC_Os06g05480 | 100 | 100 | 100 | 423 | 0.0000 | 0.0000 | 0.0010 |
| *S_1_B* | **OG-BBa0056F23.7** | Putative protein | LOC_Os06g05510 | 96.7 | 96.5 | 96.5 | 423 | 0.0032 | 0.0119 | 0.2625 |
| *S_1_B* | **OG-BBa0056F23.8** | Putative protein kinase | LOC_Os06g05520 | 99.9 | 100 | 100 | 1059 | 0.0000 | 0.0027 | 0.0010 |
| *S_1_B* | **OG-BBa0056F23.9** | Putative protein | LOC_Os06g05530 | 99.1 | 99.1 | 99.6 | 699 | 0.0041 | 0.0200 | 0.2078 |

Table S5 - Continuation

| **Locus** | ***O. glaberrima* Gene Name** | **Putative Function** | **Putative *O. sativa* (Nipponbare) orthologous gene** | **% of nucleotide identity** | **% of protein identity** | **% of similarity** | **Length (bp)** | **Ka (dN)** | **Ks (dS)** | **Ka/Ks** |
| --- | --- | --- | --- | --- | --- | --- | --- | --- | --- | --- |
| *S_1_B* | **OG-BBa0056F23.10** | Hypothetical protein | LOC_Os06g05540 | 99.1 | 98.5 | 98.5 | 582 | 0.0073 | 0.0121 | 0.6084 |
| *S_1_B* | **OG-BBa0056F23.11** | GDSL esterase/lipase protein | LOC_Os06g05550 | 99.8 | 100 | 100 | 1056 | 0.0000 | 0.0103 | 0.0010 |
| *S_1_B* | **OG-BBa0056F23.12** | Putative protein | LOC_Os06g05560 | 98.1 | 95.9 | 97.1 | 513 | 0.0213 | 0.0181 | 1.1748 |
| *S_1_B* | **OG-BBa0056F23.13** | Putative F-box protein | LOC_Os06g05580 | 99.1 | 98.2 | 98.2 | 1182 | 0.0084 | 0.0122 | 0.6896 |
| *S_1_B* | **OG-BBa0056F23.14*** | Putative F-box protein | LOC_Os06g05590 | NA | NA | NA | NA | NA | NA | NA |
| *S_1_B* | **OG-BBa0056F23.15** | Putative F-box protein | LOC_Os06g05600 | 99.5 | 99.2 | 99.5 | 1194 | 0.0033 | 0.0116 | 0.2812 |
| *S_1_B* | **OG-BBa0056F23.16** | Putative F-box protein | LOC_Os06g05610 | 98.4 | 98.1 | 98.9 | 1107 | 0.0086 | 0.0419 | 0.2053 |
| *S_1_B* | **OG-BBa0056F23.17*** | Putative F-box protein | LOC_Os06g05620 | NA | NA | NA | NA | NA | NA | NA |
| *S_1_B* | **OG-BBa0056F23.18** | GDSL esterase/lipase protein | LOC_Os06g05630 | 99.5 | 99.2 | 99.4 | 1083 | 0.0036 | 0.0090 | 0.3962 |
| *S_1_B* | **OG-BBa0056F23.19** | Putative protein | LOC_Os06g05640 | 99.5 | 98.9 | 99.2 | 1854 | 0.0053 | 0.0059 | 0.9122 |
| *S_1_B* | **OG-BBa0056F23.20** | Putative protein | LOC_Os06g05650 | 99.5 | 98.5 | 100 | 198 | 0.0075 | 0.0001 | NA |
| *S_1_B* | **OG-BBa0041E07.1** | Putative Nucleosome protein | LOC_Os06g05660 | 99.8 | 100 | 100 | 1137 | 0.0000 | 0.0118 | 0.0010 |
| *S_1_B* | **OG-BBa0041E07.2** | Putative protein | LOC_Os06g05670 | 100 | 100 | 100 | 354 | 0.0000 | 0.0000 | 0.0010 |
| *S_1_B* | **OG-BBa0041E07.3** | putative Cystein synthase protein | LOC_Os06g05690 | 99.8 | 99.7 | 100 | 1020 | 0.0013 | 0.0040 | 0.3329 |
| *S_1_B* | **OG-BBa0041E07.4** | putative Cystein synthase protein | LOC_Os06g05700 | 99.8 | 99.7 | 100 | 1044 | 0.0015 | 0.0000 | NA |
| *S_1_B* | **OG-BBa0041E07.5** | Putative protein | LOC_Os06g05710 | 98.5 | 98.3 | 98.3 | 717 | 0.0052 | 0.0266 | 0.1943 |
| *S_1_B* | **OG-BBa0041E07.6** | Putative protein | LOC_Os06g05720 | 99.8 | 99.7 | 100 | 858 | 0.0016 | 0.0048 | 0.3245 |
| *S_1_B* | **OG-BBa0041E07.7** | Putative protein | LOC_Os06g05730 | 98.5 | 98.7 | 98.7 | 672 | 0.0000 | 0.0089 | 0.0010 |
| *S_1_B* | **OG-BBa0041E07.8** | Putative protein | LOC_Os06g05740 | 99.5 | 99 | 99 | 921 | 0.0042 | 0.0105 | 0.3977 |
| *S_1_B* | **OG-BBa0041E07.9** | Putative transferase protein | LOC_Os06g05750 | 99.7 | 100 | 100 | 1425 | 0.0000 | 0.0378 | 0.0010 |
| *S_1_B* | **OG-BBa0041E07.10** | Putative ubiquitin protein | LOC_Os06g05760 | 99.7 | 97 | 98 | 915 | 0.0134 | 0.0586 | 0.2277 |
| *S_1_B* | **OG-BBa0041E07.11*** | Hypothetical protein | LOC_Os06g05770 | NA | NA | NA | NA | NA | NA | NA |
| *S_1_B* | **OG-BBa0041E07.12** | Putative transferase protein | LOC_Os06g05790 | 78.7 | 78.7 | 78.9 | 1803 | 0.0039 | 0.1038 | 0.0371 |
| *S_1_B* | **OG-BBa0041E07.13** | Putative protein | LOC_Os06g05800 | 99.6 | 100 | 100 | 903 | 0.0000 | 0.0206 | 0.0010 |
| *S_1_B* | **OG-BBa0041E07.14** | Putative trafficking protein | LOC_Os06g05804 | 99.5 | 100 | 100 | 432 | 0.0000 | 0.0135 | 0.0010 |
| *S_1_B* | **OG-BBa0041E07.15** | Putative protein | LOC_Os06g05820 | 99.6 | 99.4 | 99.5 | 1860 | 0.0032 | 0.0068 | 0.4742 |
| *S_1_B* | **OG-BBa0041E07.16** | Putative protein kinase | LOC_Os06g05830 | 98.8 | 97.7 | 97.7 | 1600 | 0.0103 | 0.0132 | 0.7807 |
| *S_1_B* | **OG-BBa0041E07.17** | Putative phosphofructokinase protein | LOC_Os06g05860 | 99.5 | 99.5 | 99.6 | 1680 | 0.0016 | 0.0109 | 0.1431 |
| *S_1_B* | **OG-BBa0041E07.18** | Putative protein phosphatase | LOC_Os06g05870 | 98.3 | 98.9 | 99.3 | 816 | 0.0051 | 0.0525 | 0.0978 |
| *S_1_B* | **OG-BBa0041E07.19** | Putative profilin protein | LOC_Os06g05880 | 99.7 | 100 | 100 | 396 | 0.0000 | 0.0167 | 0.0010 |
| *S_1_B* | **OG-BBa0041E07.20** | Putative zinc-finger protein | LOC_Os06g05890 | 98.5 | 99.4 | 100 | 1083 | 0.0027 | 0.0466 | 0.0569 |
| *S_1_B* | **OG-BBa0041E07.21** | Putative Methylase protein | LOC_Os06g05900 | 97.9 | 97.9 | 98.5 | 984 | 0.0059 | 0.0261 | 0.2250 |
| *S_1_B* | **OG-BBa0041E07.22** | Putative methyltransferases | LOC_Os06g05910 | 98.9 | 97.9 | 98.3 | 711 | 0.0104 | 0.0142 | 0.7281 |
| *S_1_B* | **OG-BBa0041E07.23** | Putative PRR protein | LOC_Os06g05920 | 92.6 | 92.9 | 93.2 | 2197 | 0.0216 | 0.0420 | 0.5148 |
| *S_1_B* | **OG-BBa0041E07.24** | Putative protein | LOC_Os06g05930 | 100 | 100 | 100 | 198 | 0.0000 | 0.0000 | NA |
| *S_1_B* | **OG-BBa0041E07.25** | Putative protein | LOC_Os06g05940 | 97.7 | 96.7 | 98 | 1650 | 0.0127 | 0.0958 | 0.1325 |
| *S_1_B* | **OG-BBa0041E07.26** | Putative protein | LOC_Os06g05950 | 97.6 | 97.2 | 98 | 1185 | 0.0115 | 0.0538 | 0.2139 |
| *S_1_B* | **OG-BBa0041E07.27** | Putative protein | LOC_Os06g05960 | 100 | 100 | 100 | 306 | 0.0000 | 0.0000 | NA |
| *S_1_B* | **OG-BBa0041E07.28** | Putative protein | LOC_Os06g05980 | 99.2 | 99.8 | 99.8 | 1296 | 0.0000 | 0.0292 | 0.0010 |
| *S_1_B* | **OG-BBa0041E07.29^p^** | / | LOC_Os06g05990 | NA | NA | NA | NA | NA | NA | NA |

p: Partial gene; *: Pseudogene; PM: Pack-MULE.
