# Supplementary material for: Patterns of Sequence Divergence and Evolution of the S1 Orthologous Regions between Asian and African Cultivated Rice Species
Source: PLoS One. 2011 Mar 10;6(3):e17726. doi: 10.1371/journal.pone.0017726 (PMC3053390; doi:10.1371/journal.pone.0017726)
Supplement: Table S6 — New molecular markers designed in the S1 regions. (DOC) [file pone.0017726.s012.doc]

Table S6 - New molecular markers designed in the *S_1_* regions

| **Molecular marker** | **Position Chr. 6 Nipponbare** | **Primer Forward** | **Primer Reverse** | **Annealing**  **(°C)** | **MgCl_2_**  **(mM)** |
| --- | --- | --- | --- | --- | --- |
| C6_19742 | 1 974 208 | TTTTCTCCTTTGGACGGAAC | TTTCTGCATGTTTGCCATTC | 61 | 1,5 |
| C6_19918 | 1 991 867 | GGCAACAACAGAGGTTTGTG | TGACCTAAATGCCCTTCTTCA | 61 | 1,5 |
| C6_20017 | 2 001 715 | ACTCGTGACCAACCCACCTA | GGGTCCCATCCTATCCAAG | 61 | 1,5 |
| RMC6_20107 | 2 010 723 | TGATTAAGTGGTGATTTGGTCA | AATGAGCATGATCACAGAGGA | 59 | 1,5 |
| RMC6_20199 | 2 019 980 | AAATCAAGGTCGGCTCATTC | TCTCGCAGAGATCAAATTGC | 59 | 1,5 |
| RMC6_20349 | 2 034 961 | TAACAGGGGGTGCTATGGAC | GGTCGAAAACGGTAGCACTT | 59 | 1,5 |
| C6_20417 | 2 041 714 | TACTACCCGACCACCTTCCT | AAACTTGTCCTTACGCTCTGTTC | 61 | 1,5 |
| C6_20707 | 2 070 780 | TCTGCAACAAGTCTGCCACT | CCTTGATCCCAAAATTGGTT | 61 | 1,5 |
| C6_20928 | 2 092 855 | ATTGGATCAACCACATGCTC | TCTGGTTGAGTTCACTGTTCAT | 61 | 1,5 |
| RMC6_21017 | 2 101 746 | TGAACTTGCACTCATTGTAAACC | GAAGTGCGCATTACAAGCTC | 59 | 1,5 |
| RMC6_21494 | 2 149 463 | CTGCTGGTGGACACATTTTG | TCATTCCAATGTCCAAACGA | 59 | 1,5 |
| C6_22972 | 2 297 236 | GAGCTTGGCGTAGCTGATTC | TTCTTCCCCATCTTCCATTG | 61 | 2,0 |
| RMC6_23181 | 2 318 116 | CATCGTGTGCCCTTCAGTTA | ACTTTAAGCCCCCATTTGGA | 59 | 1,5 |
| RMC6_23319 | 2 331 906 | TGAATGCAGCATGTGTTCCT | TCATTCTTTGCCCATTGTTTT | 59 | 1,5 |
| C6_23336 | 2 333 657 | GGCTGAATTGGGTAAGGTTG | CCCATTGGACTGCTTGTTCT | 61 | 1,5 |
| RMC6_23395 | 2 339 577 | CTGAGATTTTCTGCGTGCAA | GCGGTCATGATCAGGAAGAT | 59 | 1,5 |
| RMC6_23576 | 2 357 660 | GTCCAAGCTCACAGCCTTCT | GCATGTTTTGATGGGTGTTTC | 59 | 1,5 |
| RMC6_23679 | 2 367 997 | TCAGGAATGGGGCATAAAAC | TCCTTTTCAACTCGGTAACCA | 59 | 1,5 |
| RMC6_23801 | 2 380 193 | CCGTGTGGCAGTTTATGAGG | GAACACGCGGAGGAACAC | 59 | 1,5 |
| RMC6_23854 | 2 385 488 | TTTTTCCATGGTTGTGGGTAA | GATGCCACGATCATGCTACA | 59 | 1,5 |
| RMC6_23998 | 2 399 814 | TTTTCAAACAACATAGTTTTCGTCA | TGGCTTCTCTCGTGGAGTTT | 59 | 1,5 |
| RMC6_24382 | 2 438 297 | CCAATCCGAATAGACCCGTA | CCTCCAGGTAGTTCGGTCAA | 59 | 1,5 |
| C6_27332 | 2 733 214 | gacagtgtaaaagggacattgg | cagcctagaatttgggtgga | 61 | 1,5 |
| C6_27423 | 2 742 310 | tccgacgatatcgcttactc | ggggcagggttcacaatc | 61 | 1,5 |
| RM19398* | 2 779 675 | (IRGSP, 2005) | - | 59 | 1.5 |
| RM19400* | 2 810 860 | (IRGSP, 2005) | - | 55 | 1.5 |
| RM510* | 2 831 513 | (IRGSP, 2005) | - | 55 | 1.5 |

Marker names with the “RMC6” and “C6” prefix correspond to microsatellites and InDels respectively. (*) Primers synthesized as previously described (IRGSP, 2005. The map-based sequence of the rice genome. Nature 436: 793-800).
